# Activation of cryptic xylose metabolism by a transcriptional activator Znf1 boosts up xylitol production in the engineered *Saccharomyces cerevisiae* lacking xylose suppressor *BUD21* gene

**DOI:** 10.1186/s12934-022-01757-w

**Published:** 2022-03-05

**Authors:** Pattanan Songdech, Rawitsara Intasit, Yodying Yingchutrakul, Chutikarn Butkinaree, Khanok Ratanakhanokchai, Nitnipa Soontorngun

**Affiliations:** 1 Division of Biochemical Technology, School of Bioresources and Technology, King Mongkut’s University of Technology Thonburi, Bangkok, 10150 Thailand; 2 National Omics Center, National Science and Technology Development Agency, Pathum Thani, 12120 Thailand; 3 Pilot Plant Development and Training Institute, King Mongkut’s University of Technology Thonburi, Bangkok, 10150 Thailand

**Keywords:** Bioconversion, Metabolic engineering, Transcription factor Znf1, Xylose utilisation, Xylitol, Yeast

## Abstract

**Background:**

Xylitol is a valuable pentose sugar alcohol, used in the food and pharmaceutical industries. Biotechnological xylitol production is currently attractive due to possible conversion from abundant and low-cost industrial wastes or agricultural lignocellulosic biomass. In this study, the transcription factor Znf1 was characterised as being responsible for the activation of cryptic xylose metabolism in a poor xylose-assimilating *S. cerevisiae* for xylitol production.

**Results:**

The results suggest that the expression of several xylose-utilising enzyme genes, encoding xylose reductases for the reduction of xylose to xylitol was derepressed by xylose. Their expression and those of a pentose phosphate shunt and related pathways required for xylose utilisation were strongly activated by the transcription factor Znf1. Using an engineered *S. cerevisiae* strain overexpressing *ZNF1* in the absence of the xylose suppressor *bud21*Δ, xylitol production was maximally by approximately 1200% to 12.14 g/L of xylitol, corresponding to 0.23 g/g xylose consumed, during 10% (w/v) xylose fermentation. Proteomic analysis supported the role of Znf1 and Bud21 in modulating levels of proteins associated with carbon metabolism, xylose utilisation, ribosomal protein synthesis, and others. Increased tolerance to lignocellulosic inhibitors and improved cell dry weight were also observed in this engineered *bud21*∆ + pLJ529-*ZNF1* strain. A similar xylitol yield was achieved using fungus-pretreated rice straw hydrolysate as an eco-friendly and low-cost substrate.

**Conclusions:**

Thus, we identified the key modulators of pentose sugar metabolism, namely the transcription factor Znf1 and the suppressor Bud21, for enhanced xylose utilisation, providing a potential application of a generally recognised as safe yeast in supporting the sugar industry and the sustainable lignocellulose-based bioeconomy.

**Graphical Abstract:**

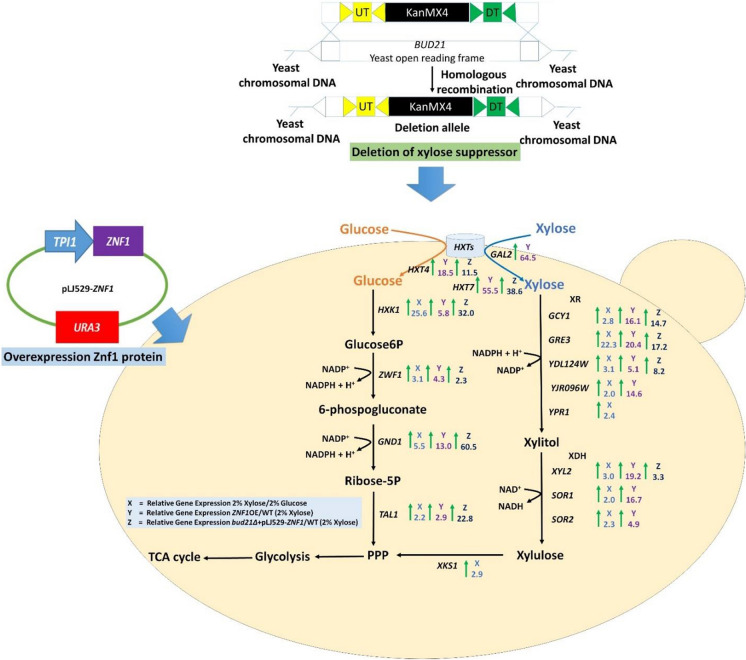

**Supplementary Information:**

The online version contains supplementary material available at 10.1186/s12934-022-01757-w.

## Background

Xylitol has been identified as one of the top 12 value-added compounds to be attained from biomass [1]. It is a natural sweetener used as a sugar substitute in the food and pharmaceutical industries. It offers advantageous properties as a low-calorie sweetener with insulin-independent metabolism and enables the control of calorie intake of consumers [2]. Furthermore, xylitol can be used in the biomedical industry as an intermediate for the synthesis of biodegradable polymers [3]. Owing to these properties, demand for xylitol by the food and pharmaceutical industries has increased. The worldwide market of xylitol is estimated to be more than 700 million tons per year, and its selling price is approximately 4–5 US dollars per kilogram, making it more expensive than other sugars [4]. Currently, xylitol is industrially produced through the chemical hydrolysis of xylan and xylose-hydrogenation reactions. Alternatively, the use of lignocellulose as a raw material has resulted in growing interest in the microbiological production of xylitol. This method is more environmentally friendly than the chemical method, which requires expensive equipment, extensive purification steps, and huge energy demand [5].

Several yeasts are naturally capable of consuming xylose through a xylose reductase/xylitol dehydrogenase (XR/XDH) pathway for the conversion of xylose into xylitol and subsequently to xylulose. Xylitol is a metabolic intermediate and by-product of this pathway, mainly resulting from the co-factor imbalance between the reaction catalysed by the NAD(P)H-dependent xylose reductase with a preference for NADPH and the NAD^+^-dependent xylitol dehydrogenase [2]. For example, strains of native xylose-utilising yeast *Pichia stipitis* or *Scheffersomyces stipitis* (CBS 5773, 5774, 5775, 5776) produce xylitol at 0.05–0.19 g/g xylose consumed using 30 g/L xylose, pH 5.0, in anaerobic conversion experiments [6]. *Hansenula polymorpha* ATCC 34438 also produces xylitol at 12 g/L or 0.61 g/g xylose consumed, when using 20 g/L xylose after 75 h of fermentation [7]. *Kluyveromyces marxianus* IMB5 produces xylitol at 1.3 g/L or 0.25 g/g from 20 g/L of xylose after 48 h of fermentation [8]. *Meyerozyma guilliermondii* produces a high yield of 40.40 g/L xylitol or 0.49 g/g of consumed xylose from 100 g/L xylose after 10 days of fermentation [9]. Naturally xylose-assimilating yeasts have been extensively studied during glucose–xylose co-fermentation for xylitol production. During co-fermentation, *Debaryomyces hansenii* and *Candida guilliermondii* produce xylitol from rapeseed straw detoxified hydrolysates after 144 h at 0.45 g/g xylose consumed [10]. *Kluyveromyces marxianus* (CCA 510) produces xylitol at 6.76 g/L, after 96 h of cashew apple bagasse hydrolysate fermentation [11]. *Candida guilliermondii* also produces 32.7 g/L of xylitol after 48 h of fermentation of a eucalyptus hemicellulosic hydrolysate [12]. Nevertheless, the yields of xylitol produced from xylose are still limited because xylose is also used for cell metabolism and energy maintenance; moreover, some yeast species are considered unsafe for use in the food industry such as *P. stipites,* and *M. guilliermondii* [13].

The yeast *S. cerevisiae*, a generally recognised as safe microorganism, is naturally incapable of efficient xylose metabolism. Currently, there are two major successfully implemented strategies for xylose utilisation by *S. cerevisiae* via the oxidoreductase (XR/XDH) pathway and the xylose isomerase (XI) pathway. The oxidoreductase strategy uses a xylose reductase (XR) that reduces xylose to xylitol, preferably using NADPH over NADH as the cofactor, and a xylitol dehydrogenase (XDH) that uses only NAD^+^ as the cofactor to convert xylitol to xylulose [14]. The XR/XDH pathway utilisation has a major bottleneck caused by the issue of cofactor imbalance between the NADPH-dependent XR- and the NAD^+^-dependent XDH-reactions, causing the accumulation of xylitol and thus lowering ethanol production [15]. Secondly, the isomerase pathway, which is mainly found in bacteria, is a one-step reaction catalysed by XI. This enzyme directly converts xylose to xylulose without requiring cofactors [16, 17]. However, XI activity is inhibited by xylitol formation by the unspecific endogenous NADPH-dependent aldose reductase (*GRE3*) gene product that converts xylose to xylitol [18]. Additionally, overexpression of genes involved in the non-oxidative pentose phosphate (PPP) shunt and deletion of the *GRE3* gene improve xylose utilisation in strains expressing the xylose-utilising pathway [19].

The ability of some industrial yeasts to utilise xylose has been mapped to a putative xylitol dehydrogenase gene, *XDH1*, which is not present in the laboratory *S. cerevisiae* S288C strain [20]. Xylitol dehydrogenase activity requires the endogenous XR genes *GRE3* and *YPR1* and the endogenous *XKS1* gene to allow for xylose utilisation. Three putative XDH genes, i.e. *SOR1*, *SOR2*, and *XYL2*, can suppress the ability of *XDH1-*expressing strains to utilise xylose, as shown by increased expression of enzymes with xylose reductase activity. This has emerged as a potential solution to increase xylitol yields from xylose [20, 21]. Additionally, overexpression of genes involved in the non-oxidative pentose phosphate (PPP) shunt and deletion of the *GRE3* gene also improve xylose utilisation in strains expressing the xylose-utilising pathway [19]. Surprisingly, synthetic genomic array (SGA) identified genes whose modification affects xylose consumption, including *BUD21*, encoding a less well-characterised component of the small ribosomal subunit [22]. When these genes were individually deleted, xylose utilisation was improved in both *S. cerevisiae* S288C and CEN.PK strains [22]. Importantly, the *BUD21* deletion strain, characterised in batch fermentation, was found to produce increased levels of xylitol (1.35 g/L) and ethanol (3.19 g/L) even in the absence of exogenous XI (*xylA*), demonstrating the suppressor ability of Bud21 in a laboratory *S. cerevisiae* strain utilising xylose as a sole carbon source [22]. This finding implies that *S. cerevisiae* may not require the addition of exogenous genes for xylose fermentation.

Previous studies on xylose fermentation by yeasts have revealed that some non-recombinant *S. cerevisiae* strains could slightly grow on xylose as a sole carbon source [23]. Despite the presence of all transporters and enzymes required for xylose utilisation in the genome of *S. cerevisiae*, their expression is just too low to ensure efficient xylose fermentation [24]; therefore, engineering exogenous genes of the xylose-utilising pathway has been the mainstream method to improve the xylose fermentation efficiency of *S. cerevisiae*. Despite several attempts to engineer xylose fermenting *S. cerevisiae* strains, the xylose metabolic pathway does not enable the yeast to rapidly and effectively utilise xylose. Several limitations still need to be overcome, including glucose repression, slow xylose transport, and cofactor imbalance in the xylose reductase/xylitol dehydrogenase pathway [25]. Alternatively, the approach that focuses on identifying and optimising the endogenous xylose-utilisation pathway in this yeast needs to be intensively investigated [22].

The role of the transcription factor Znf1 in alternative carbon source utilisation during the glucose-ethanol shift has been reported and is implicated in non-fermentable carbon source utilisation and stress responses [26, 27]. Recently, Znf1 was shown to be an activator of genes in glycolysis and alcoholic fermentation during high glucose fermentation [28]. Its overexpression leads to increased ethanol productivity and enhances tolerance to osmotic and weak-acid stresses [28]. A previous study reported that *ZNF1* knockout strongly decreases xylose utilisation during the post-glucose effect, but its overexpression had no effect [29]. Additionally, in another study, the overexpression or deletion of the *ZNF1* gene did not affect the fermentation ability of a xylose-fermenting strain engineered on the *S. cerevisiae* GS010 background [30]. Nevertheless, the role of Znf1 in xylose utilisation in native *S. cerevisiae* has never been fully explored. In this study, the aims were to investigate the transcriptional control of genes related to xylose metabolism, PPP, glycolysis, and the fermentative pathway governed by Znf1 and, secondly, to construct an *S. cerevisiae ZNF1-*overexpressing strain on different backgrounds with or without the Bud21 suppressor for co-fermentation of low glucose–xylose or agricultural waste for potential xylitol production. The possibility of engineered *S. cerevisiae* strains to circumvent a major barrier that limits effective xylose utilisation from lignocellulosic biomass was investigated using gene and protein expression studies with batch fermentation.

## Results

### Rewiring xylose metabolism and related pathways during the glucose–xylose shift

**Fig. 1:**
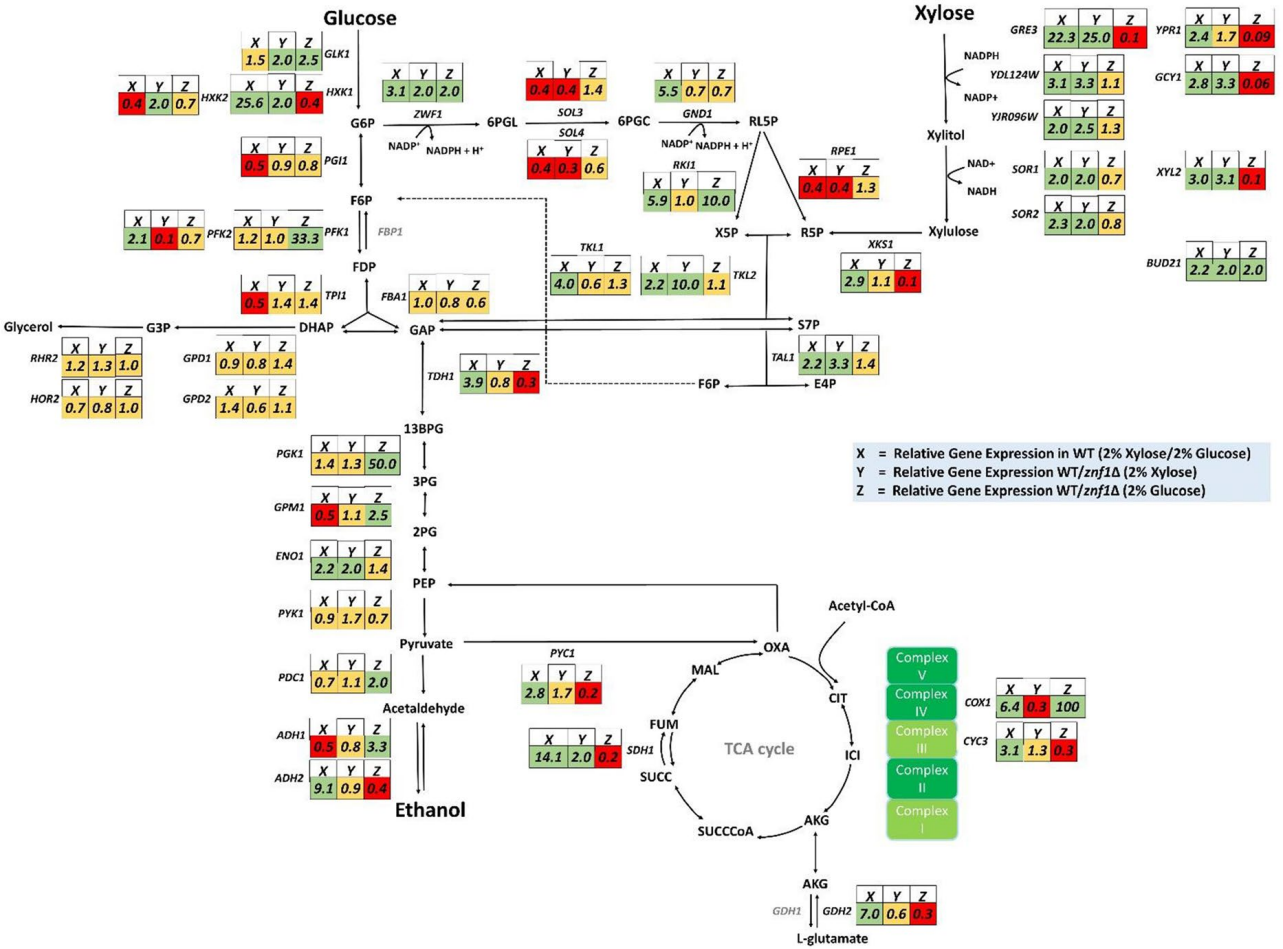
Expression levels of Znf1 target genes during glucose–xylose shift. Relative expression levels of genes involved in xylose metabolism, pentose phosphate pathway, glycolysis, gluconeogenesis, glycerol metabolism and TCA cycle were examined using RT-qPCR. Changes in the levels of mRNAs were indicated as following: 2% xylose shift in the wild-type strain (the X rectangle box), in the wild-type strain relative to the *znf1*∆ strain during the 2% xylose shift (the Y rectangle box) and, in the wild-type strain relative to the *znf1*∆ strain during growth in 2% glucose (the Z rectangle box). Green, red or yellow color box indicated genes whose expression was either activated/induced, repressed or unaltered by xylose or the transcription factor Znf1, respectively. The relative expression levels were obtained via the comparative C_t_ method for quantification of the ∆∆C_t_ values. Altered expression more than 2-folds was considered significant. The arrowheads in the figure represent the direction of enzymatic reactions. Abbreviations: *ADH1*, alcohol dehydrogenase I; *ADH2*, alcohol dehydrogenase II; *BUD21*, component of small ribosomal subunit/ xylose suppressor; *COX1*, cytochrome c oxidase; *CYC3*, cytochrome c heme lyase; *ENO1*, enolase; *FBA1*, fructose 1,6-bisphosphate aldolase; *GCY1*, glycerol dehydrogenase; *GDH2*, glutamate dehydrogenase; *GLK1*, glucokinase; *GND1*, 6-phosphogluconate dehydrogenase; *GPD1*, glycerol-3-P dehydrogenase; *GPD2*, glycerol-3-P dehydrogenase; *GPM1*, phosphoglycerate mutase; *GPP1*, glycerol-3-P phosphatase; *GPP2*, glycerol-3-P phosphatase; *GRE3*, aldose reductase; *HXK1*, hexokinase isoenzyme 1; *HXK2*, hexokinase isoenzyme II; *PFK1*, phosphofructokinase I; *PFK2*, phosphofructokinase II; *PGI1*, phosphoglucose isomerase; *PGK1*, 3-phosphoglycerate kinase; *PDC1*, pyruvate decarboxylase; *PYC1*, pyruvate carboxylase; *PYK1*, pyruvate kinase; *RKI1*, ribose-5-phosphate ketol-isomerase; *RPE1*, ribulose-5-phosphate 3-epimerase; *SDH1*, succinate dehydrogenase; *SOL3*, 6-phosphogluconolactonase; *SOL4*, 6-phosphogluconolactonase; *SOR1*, sorbitol dehydrogenase; *SOR2*, sorbitol dehydrogenase; *TAL1*, transaldolase; *TDH1*, glyceraldehyde-3-phosphate dehydrogenase; *TKL1*, transketolase; *TKL2*, transketolase; *TPI1,* triose phosphate isomerase; *XKS1*, xylulokinase; *XYL2*, xylitol dehydrogenase; *YDL124W*, NADPH-dependent alpha-keto amide reductase; *YJR096W*, xylose and arabinose reductase; *YPR1*, NADPH-dependent aldo–keto reductase; *ZWF1*, glucose-6-phosphate dehydrogenase. Compounds, AKG, α-ketoglutarate; 13BPG, 1,3-bisphosphoglycerate; CIT, citrate; DHAP, dihydroxyacetone phosphate; E4P, erythrose 4-phosphate; FDP, fructose-1,6-diphosphate; F6G, fructose-6-phosphate; FUM, fumarate; GAP, glyceraldehyde-3-phosphate; G6P, glucose-6-phosphate; G3P, glyceraldehyde-3-phosphate; ICI, isocitrate; PEP, phosphoenolpyruvate; 3PG, 3-phosphoglycerate; 6PGC, 6-phosphogluconate; 6PGL, 6-phospho-gluconolactonase; MAL, malate; OXA, oxaloacetate; R5P, ribose 5-phosphate; RL5P, ribulose 5-phosphate; S7P; sedoheptulose 7-phosphate; SUCCoA, succinyl CoA; SUCC, succinate; X5P, xylulose 5-phosphate

In *S. cerevisiae*, utilisation of poor carbon sources such as xylose induces a starvation response and increases the expression of adaptive stress-response genes [31]. During growth in xylose, the expression of genes involved in xylose metabolism was significantly up-regulated when compared with growth under glucose conditions in the wild-type BY4742 strain [32] (Additional file 1: Table S1 and Fig. 1). The observed induction included multiple aldo–keto reductase genes for the conversion of xylose to xylitol, namely *GRE3* (22.3-fold), *YPR1* (2.4-fold), *GCY1* (2.8-fold), *YDL124W* (3.1-fold), and *YJR096W* (2.0-fold), as well as xylitol dehydrogenase genes for the conversion of xylitol to xylulose, including *XYL2* (3.0-fold), *SOR1* (2.0-fold), and *SOR2* (2.3-fold), and a xylulokinase gene *XKS1* (2.9-fold) for the conversion of D-xylulose and ATP to xylulose 5-phosphate and ADP (Additional file 1: Table S1 and Fig. 1). Thus, in the absence of glucose, even for the native yeast *S. cerevisiae,* xylose could effectively induce the expression of endogenous genes required for the metabolism and utilisation of xylose as a sole carbon source. Interestingly, the expression of the small *BUD21* gene, known to repress xylose utilisation, was also up-regulated (2.2-fold) during growth in xylose in the wild-type strain (Additional file 1: Table S1 and Fig. 1). In agreement, previous RNA-seq data has indicated that the *S. cerevisiae* wild-type Y133 strain shows significantly increased in mRNA levels of *ZNF1* and *BUD21* during anaerobic xylose growth [33], supporting their major role as a transcription factor and modulator of xylose utilisation, respectively.

Additionally, the transcriptional control of genes involved in gluconeogenesis, glycolysis, and the pentose phosphate pathway (PPP), was also investigated during growth in xylose, as these metabolic pathways are tightly interconnected with xylose utilisation. In the wild-type *S. cerevisiae*, the expression of gluconeogenic genes, namely *HXK1* (25.6-fold), *TDH1* (3.9-fold)*, ENO1* (2.2-fold)*, PYC1* (2.8-fold), and *ADH2* (9.1-fold), was also up-regulated during the glucose–xylose shift (Additional file 1: Table S1 and Fig. 1). This suggested the ability of this strain to utilise a C5 carbon for the synthesis of glucose-6-phosphate, a key metabolic precursor providing the RNA backbone precursors ribose 5-phosphate and erythrose 4-phosphate for aromatic amino acid production important for cell proliferation and survival [34, 35]. In fact, gluconeogenesis is a key pathway for cells to drive the flux though acetyl-CoA, a key substrate in the tricarboxylic acid (TCA) cycle important for basic cellular functions such as energy supply as well as lipid and amino acid metabolism [36]. On the other hand, the expression of *PGI1, TPI1* and *GPM1*, components of the glycolysis pathway, as well as *ADH1* involved in alcohol fermentation, was down-regulated by approximately twofold during growth on xylose as the sole carbon source; in glucose-grown cells, expression of these genes was de-repressed by approximately twofold (Additional file 1: Table S1 and Fig. 1), suggesting the rewiring of carbon metabolism. The expression of genes involved in glycerol biosynthesis (*GPD1/2* and *GPP1/2*) remained unchanged, while the expression of TCA cycle genes including *COX1* (6.4-fold), *CYC3* (3.1-fold), and *GDH2* (7.0-fold) was strongly up-regulated during xylose induction in the wild-type strain (Additional file 1: Table S1 and Fig. 1). This suggests that glycolysis flux is low and the metabolism of cells appears to be shifted from fermentation to respiration mode, as also found during glucose depletion [37]. During the transition from glucose to xylose, many genes in the PPP, such as *ZWF1* (3.1-fold), *RKI1* (5.9-fold), *GND1* (5.5-fold), *TAL1* (2.2-fold), and *TKL2* (2.2-fold), were also up-regulated while the expression of *SOL3/4* and *RPE1* was down-regulated (2.0-fold) in the wild-type *S. cerevisiae* following xylose induction (Additional file 1: Table S1 and Fig. 1). Previously, overexpression of *ZWF1* was shown to drive metabolic flux from glucose into the PPP to increase xylose utilisation during simultaneous xylose and glucose fermentation [38]. Moreover, the oxidative PPP and gluconeogenesis are enhanced during anaerobic xylose growth, possibly driven by increased demand for the cofactor NADPH [39].

### The transcription factor Znf1 actively up-regulated the expression of genes involved in xylose utilisation

Since the transcription factor Znf1 is activated during glucose de-repression and is responsible for the up-regulated expression of several genes required for utilisation of alternative carbon sources [26], its role in xylose metabolism was addressed. First, in the wild-type glucose grown cells, Znf1 was shown to activate some genes in glycolysis and the alcohol dehydrogenase *ADH1* gene, but no significant alteration of *PGI1*, *FBA1,* and *ENO1* expression was observed (Additional file 1: Table S1 and Fig. 1). During the glucose–xylose shift, expression of xylose utilisation genes was dramatically up-regulated in the wild-type strain, compared to the *znf1*Δ strain *S. cerevisiae* BY4742, including xylose reductase genes such as *GRE3* (25.0-fold), *GCY1* (3.3-fold), *YDL124W* (3.3-fold), and *YJR096W* (2.5-fold), xylitol dehydrogenase genes such as *XYL2* (3.1-fold), *SOR1* (2.0-fold), and *SOR2* (2.0-fold), as well as the xylose suppressor gene *BUD21* (2.0-fold) (Additional file 1: Table S1 and Fig. 1). These results are consistent with previous findings that the *GRE3* and *SOR1* genes of *S. cerevisiae* are capable of providing xylose reductase and xylitol dehydrogenase functions, thereby permitting cell growth on xylose as a sole carbon source [40]. Overall, in addition to non-fermentable carbon utilisation, gene expression analysis indicated that Znf1 is a key transcription regulator of xylose metabolism in *S. cerevisiae*.

In 2% xylose, the expression of some PPP genes was down-regulated in the wild-type strain by 2 to threefold when compared to the *znf1*Δ strain, such as *SOL4*, *SOL3*, and *RPE1*, suggesting a repressor role of Znf1 to block unnecessary expression of genes in the oxidative PPP branch. In contrast, *ZNF1* dramatically activated the expression of *TKL2* (10.0-fold), *TAL1* (3.3-fold), and *ZWF1* (2.0-fold) as compared to *ZNF1* deletion strain (Additional file 1: Table S1 and Fig. 1), suggesting an activator role to promote the metabolism of pentose sugars such as xylose. These genes are important for generating key metabolites that are the main building blocks for the biosynthesis of nucleic acids, and aromatic amino acids, NADPH generation, and oxidative stress responses [41, 42]. Increased expression of *TKL2, TAL1,* and *ENO1* by Znf1 during xylose utilisation may lead to a superior xylose-utilisation capability in the yeast strain. Recently, highly activated transketolase and transaldolase activity and their complex interactions in the non-oxidative PPP branch were found to be critical for serial sugar transformation to drive metabolic flow into glycolysis, which increases ethanol production [43]. Interestingly, in xylose, Znf1 mediated the activation of genes involved in lower glycolysis, which are responsible for driving the flux from phosphoenolpyruvate [44] to pyruvate and eventually to oxaloacetate (Additional file 1: Table S1 and Fig. 1). Up-regulated expression of the *SDH1* gene of the TCA cycle (2.0-fold) (Additional file 1: Table S1 and Fig. 1), suggesting the rewiring of gene expression required for oxidative metabolism. These results indicate Znf1’s control of metabolic flux toward the TCA cycle instead of ethanol formation, which is more beneficial to yeast cells during growth on a poor carbon source. Enhanced expression of genes involved in glycolysis was found in the xylose-fermenting recombinant strain during the exponential growth phase of mixed sugar fermentation.

### Overexpression of *ZNF1* increased xylose transport and metabolism

**Fig. 2:**
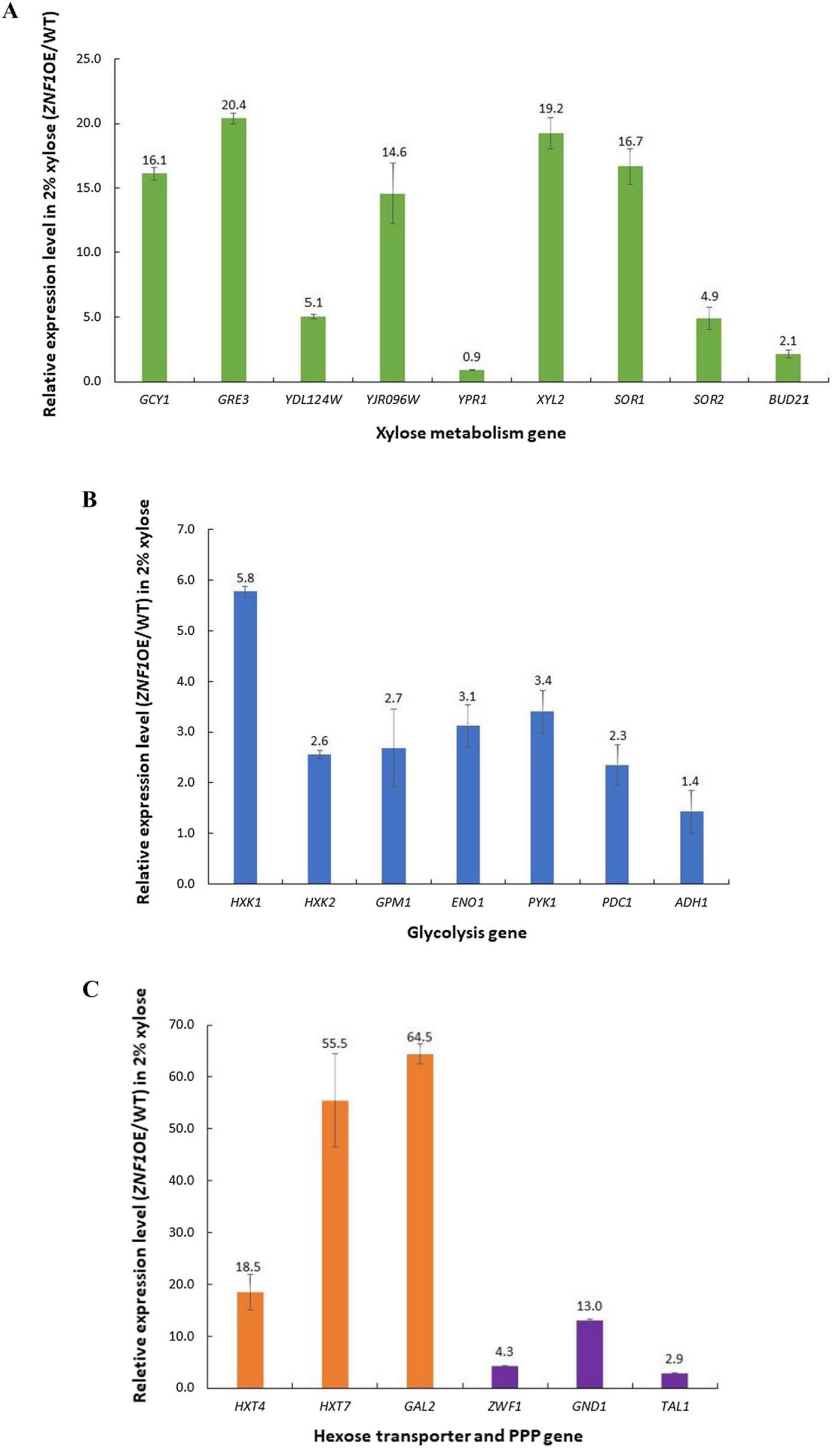

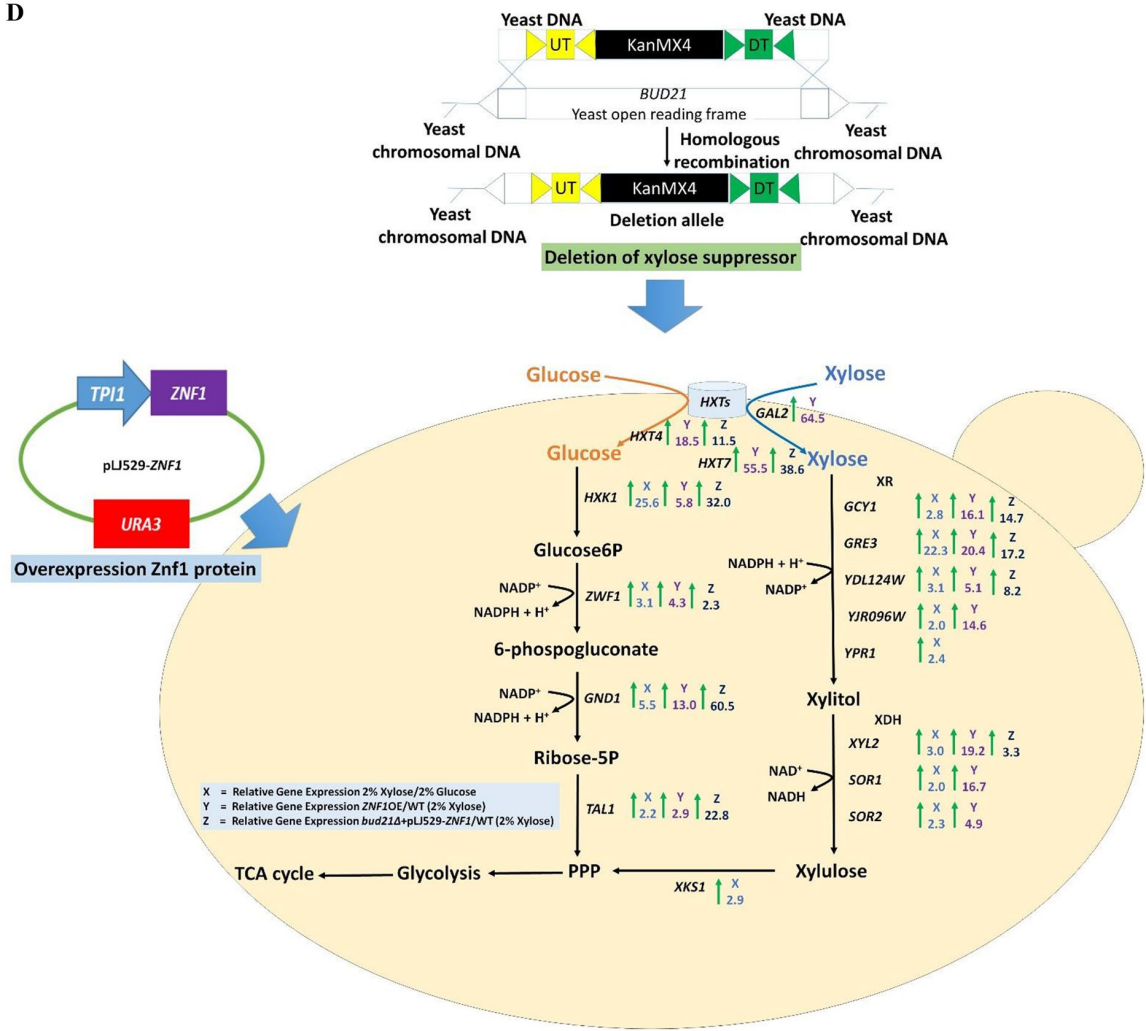
Expression levels of genes involved in xylose metabolism in the *ZNF1* overexpressing strain (*ZNF1*-OE) during the growth in 2% xylose and 0.05% glucose mix. **A** Relative expression levels of xylose metabolic genes (*GCY1, GRE3, YDL124W, YPR1, XYL2, SOR1, SOR2* and *BUD21* genes) **B** glycolytic and alcoholic fermentative genes (*HXK1, HXK2, GPM1, ENO1, PYK1, PDC1,* and *ADH1* genes) **C** hexose transporter and PPP genes (*HXT4, HXT7*, *GAL2, ZWF1, GND1,* and *TAL1* genes) in *ZNF1*-OE strain compared to the wild type strain during the growth in 2% xylose and 0.05% glucose mix. The relative expression levels were obtained via the comparative C_t_ method for quantification of the ∆∆C_t_ values. Altered expression levels more than 2-folds were considered significant. The average values were calculated from at least two independent experiments performed in three replicates. **D** Metabolic engineering strategy via overexpression of *ZNF1* transcription factor gene to activate its target genes linked to xylose metabolism and deletion of xylose suppressor *BUD21* in *S. cerevisiae*. The green arrow indicated induction of genes expressed by fold-changes which is compared to the wild-type BY4742 strain

A *ZNF1*-overexpressing strain (*ZNF1*-OE) was constructed using the CRISPR/Cas9 gene editing technique and its expression was increased 6.8-fold when compared to the wild-type strain BY4742. Gene expression of xylose reductases, targets of Znf1, was also strongly up-regulated in this engineered strain when compared to the wild-type strain, including *GCY1* (16.1-fold), *GRE3* (20.4-fold), *YDL124W* (5.1-fold), and *YJR096W* (14.6-fold) (Fig. 2A). Likewise, expression of xylitol dehydrogenase genes, including *XYL2* (19.2-fold), *SOR1* (16.7-fold), *SOR2* (4.9-fold) and also *BUD21* (2.1-fold) (Fig. 2A), as well as genes involved in the upper glycolysis pathway such as *HXK1* (5.8-fold), and *HXK2* (2.6-fold), lower glycolysis genes *GPM1* (2.7-fold), *ENO1* (3.1-fold), and *PYK1* (3.4-fold), the fermentation gene *PDC1* (2.3-fold) (Fig. 2B) and PPP genes including *ZWF1* (4.3-fold), *GND1* (13.0-fold), and *TAL1* (2.9-fold) (Fig. 2C) were highly increased in the *ZNF1*-overexpressing strain compared with the wild-type strain during growth in xylose. The xylose transporter is also known as a major bottleneck in xylitol production due to the lack of a specific pentose transporter in *S. cerevisiae*. Pentose sugars enter the cell with low affinity via glucose transporters of the Hxt family. Under low glucose conditions, xylose transport has been shown to be mediated through high-affinity glucose transporters. In the *ZNF1*-overexpressing strain, expression of *HXT4* (18.5-fold), *HXT7* (55.5-fold), and *GAL2* (64.5-fold) were increased in xylose (Fig. 2C), suggesting their important role as xylose-transporting proteins.

The overview of Znf1-mediated xylose utilisation and its target genes was summarised (Fig. 2D). Like glucose, xylose also enters yeast cells via hexose transporters whose expression is strongly induced and dependent on Znf1. Then, xylose is converted to xylitol via several xylose reductases whose expression is also under the control of the transcription factor Znf1. Xylitol is subsequently converted to xylulose via many xylitol dehydrogenases, which all are targets of Znf1. Then, it is converted to xylulose 5-phosphate and ADP by xylulokinase, whose expression is not dependent on Znf1, prior to entering the PPP pathway. As shown in the present study, xylose induced the expression of key PPP genes, namely *TAL1*, *GND1*, and *ZWF1* for the formation of the cofactor NADPH and glucose-6-phosphate. The expression of these genes was also positively regulated by Znf1, except for *GND1*. Since Znf1 chiefly modulates xylose metabolism, overexpression of *ZNF1* was speculated to improve xylose utilisation and thus the production of xylose-derived metabolites, including xylitol. To further improve xylose fermentation, deletion of the xylose suppressor *BUD21*, known to increase xylose utilisation [22] and positively regulated by Znf1, was carried out to construct the *bud21*∆ + pLJ529-*ZNF1* strain. During xylose induction, the expression of genes encoding hexose transporters (*HXT4*, 11.5-fold; *HXT7*, 38.6-fold), xylose reductase (*GCY1*, 14.7-fold; *GRE3*, 17.2-fold; *YDL124W*, 8.2-fold), xylitol dehydrogenase (*XYL2*, 3.3-fold), and PPP components (*TAL1*, 22.8-fold; *GND1*, 60.5-fold; *ZWF1*, 2.3-fold) was dramatically increased in the *bud21*∆ + pLJ529-*ZNF1* strain when compared to the wild-type strain (Fig. 2D).

### *ZNF1* overexpression and *BUD21* deletion increased growth and xylose utilisation in *S. cerevisiae*

**Fig. 3:**
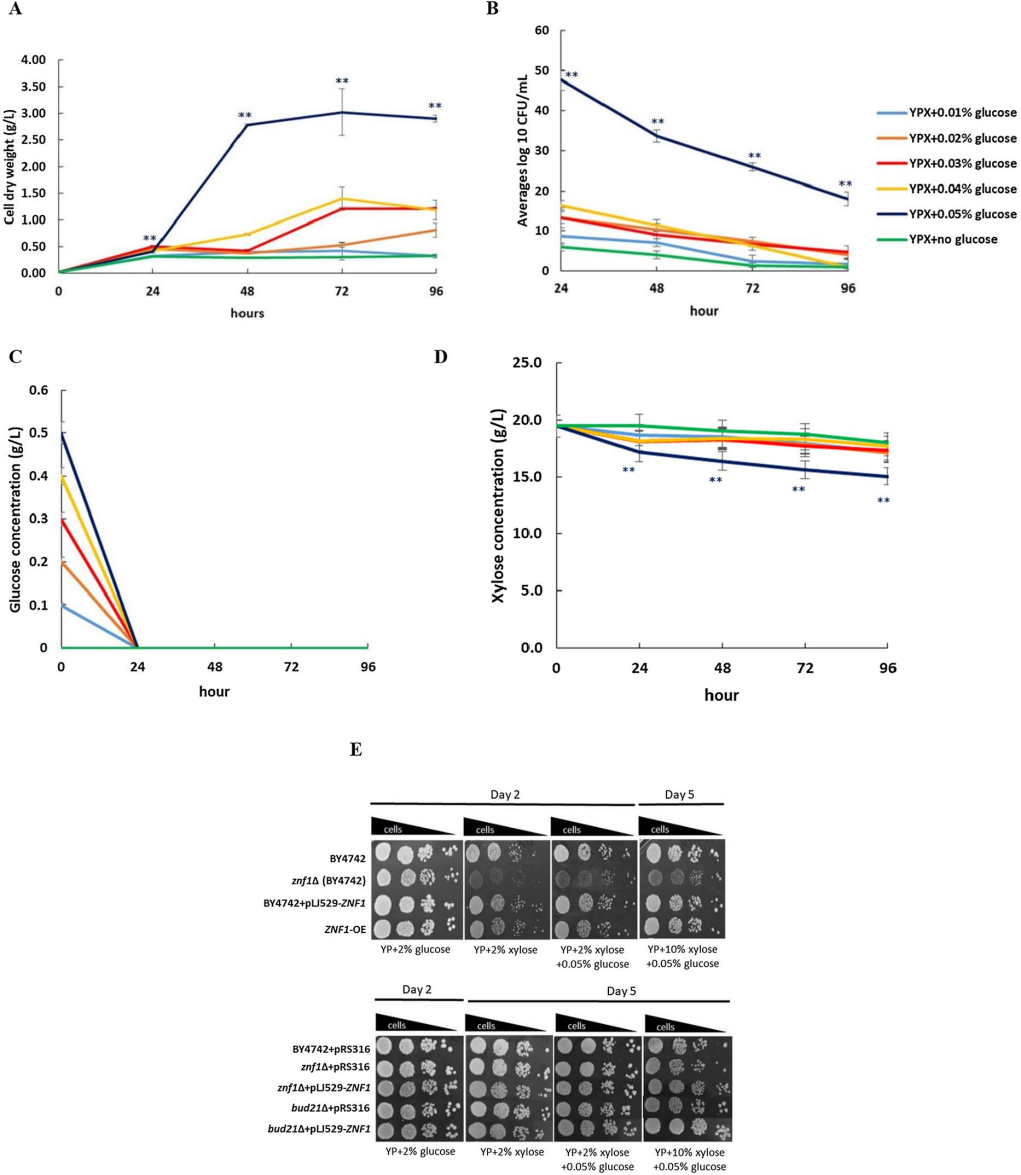
Effect of low glucose–xylose mix on induction of xylose utilization in different *S. cerevisiae* strains. The *ZNF1*-OE strain overexpressing *ZNF1* gene was investigated during culture in YPX containing 2% xylose (w/v) and low glucose at concentration of 0.05%, 0.04%, 0.03%, 0.02%, 0.01% (w/v) or without glucose expressed in term of cell dry weight (g/L) (**A**), Cell survival was analyzed using CFU/ml method (**B**), Glucose concentration (g/L) (**C**), Xylose concentration (g/L) (**D**), Phenotypic analysis on increased concentration of xylose (**E**). The wild-type BY4742, *the znf*1∆, *the bud21*∆, the rescued strain (*znf*1∆ + pLJ529-*ZNF1*) the overexpression *ZNF1* (BY4742 + pLJ529-*ZNF1* and *ZNF1*-OE)*,* and the engineered *bud21*∆ + pLJ529-*ZNF1* of *S. cerevisiae* strains were observed on YPX agar plates contained 0.05% glucose mixed with different concentration of xylose at 2 or 10% (w/v). Ten-fold serial dilutions of cells were spotted on plates and incubated at 30 °C for 2–5 days. Error bars indicated standard deviations calculated from at least two independent experiments performed in triplicate. Significance differences were determined by one-way ANOVA with Tukey HSD method (*, p < 0.05; **, p < 0.01)

Different approaches have been used to enhance xylose transport, including co-fermentation of xylose with other carbon sources [45]. In this study, the *ZNF1*-overexpressing strain was tested for increased xylose utilisation in at mixed concentrations of low glucose and 2% (w/v) xylose substrate. Interestingly, glucose at 0.05% (w/v) was the best concentration for growth when compared with the other glucose concentrations tested (Fig. 3A). At lower glucose concentrations of 0.01–0.04% (w/v), the final cell dry weights were increased by 2–260% after 48 h of fermentation with extended log phases of growth (Fig. 3A). The duration of the lag phase was significantly shorter using 0.05% (w/v) glucose, and the highest cell dry weight was obtained at approximately 3.0 g/L, i.e. 778.21% increase (Fig. 3A). On the other hand, under no glucose conditions, the lag phase time was extended to 96 h or more with no significant increase in cell biomass observed (Fig. 3A). Furthermore, colony-forming unit (CFU) assays were conducted to examine cell survival, which may not have obviously been observed by the measurement of optical density. At 24 h of incubation under 2% xylose mixed with 0–0.05% glucose, the *ZNF1*-OE strain showed dramatically increased cell colony-forming units (CFU/mL) at 47.67 × 10^7^ as compared to growth without glucose (CFU/mL of 6.00 × 10^7^), or 0.04% glucose (CFU/mL of 16.33 × 10^7^) (Fig. 3B). Moreover, glucose consumption was completed within 24 h under the low percentage glucose concentrations tested, while the *ZNF1*-OE strain quickly consumed xylose under 2% xylose + 0.05% glucose (Fig. 3C). Also, the xylose was consumed relatively quickly during the first 24 h and slightly decreased thereafter (Fig. 3D). Overall, these results demonstrate the effect of co-substrate utilisation between low glucose and xylose in the activation of xylose consumption.

Spot assays in agar YP plates containing 2% or 10% (w/v) xylose mixed with 0.05% (w/v) glucose were also conducted to confirm the involvement of Znf1 and Bud21 in xylose utilisation and osmotic stress tolerance (Fig. 3E). Growth of the *znf1*Δ strain under a higher xylose concentration was impaired as compared to the wild-type strain (Fig. 3E). The *S. cerevisiae ZNF1*-overexpressing strain showed a similar pattern of cell survival as the wild-type strain in the spot test (Fig. 3E). The *bud21*∆ strain showed slightly increased cell survival when compared to the wild-type control, while the effect was further enhanced in the *bud21*∆ + pLJ529-*ZNF1* strain (Fig. 3E), suggesting that combined genetic manipulation is more effective for increased cell survival. Importantly, the co-substrate of 0.05% (w/v) glucose and xylose was essential and thus selected for subsequent investigations into xylose utilisation and xylitol production in the engineered *S. cerevisiae* strains.

### *ZNF1* overexpression and *BUD21* deletion increased xylose to xylitol production

The general cellular response to alternative carbon sources includes an alteration in gene expression, which consequently reflects changes in metabolomic profiles. For example, the transition from glucose to xylose results in increased concentrations of amino acids and TCA-cycle intermediates and decreased concentrations of sugar phosphates and redox cofactors [46]. The up-regulated expression of genes in the PPP are likely responsible for the observed alterations. Next, the metabolite profiles of the engineered *S. cerevisiae* strains during xylose fermentation were investigated at high xylose concentrations, i.e. 2% or 10% (w/v) xylose mixed with 0.05% (w/v) glucose (Table 1). The expression level of *ZNF1* in the plasmid form of the BY4742 + pLJ529-*ZNF1* strain was increased by approximately 13-fold as compared to the control BY4742 + pRS316 strain. It was also higher than that of the integrated *ZNF1* strain, previously generated using the CRISPR technique (7.0-fold) [47]. Thus, the plasmid-derived *ZNF1*-overexpressing strain was chosen for further experiments to measure xylose utilisation and xylitol production. Notably, the *ZNF1* gene was expressed under the strong TPI promoter in the BY4742 + pLJ529-*ZNF1* and *bud21*∆ + pLJ529-*ZNF1* strains. The pRS316 plasmid was used as the empty vector, containing the *URA3* gene as a selection marker, which affects cell growth. Therefore, growth and metabolite production was compared between strains that had undergone similar genetic manipulation using at least three different replicates from two independent experiments.

**Table 1:** Fermentation profiles of engineered *S. cerevisiae* strains during growth in YP media containing 0.05% glucose (w/v) mixed with 2% xylose or 10% xylose* (w/v) at day 8 or day 14* of fermentation which provide maximal xylitol concentrations, respectively

Parameter/Strains	Day	BY4742 + pRS316	BY4742 + pLJ529 − *ZNF1*	*znf1*∆ + pRS316	*bud21*∆ + pRS316	*bud21*∆ + pLJ529 − *ZNF1*
Initial DCW (g/L)	0	0.23	0.23	0.23	0.23	0.23
Final DCW (g/L)	Day 8 Day 14*	0.99 ± 0.02 1.30 ± 0.01	0.95 ± 0.06 1.16 ± 0.02	0.53 ± 0.03 0.88 ± 0.04	1.67 ± 0.08 1.47 ± 0.03	2.19 ± 0.06 3.14 ± 0.03
Biomass specific rates (g/L/h)	Day 8 Day 14*	0.005 0.004	0.005 0.003	0.003 0.003	0.009 0.004	0.011 0.009
Xylose residues (g/L)	Day 8 Day 14*	15.71 ± 0.02 57.13 ± 0.53	14.70 ± 0.04 49.90 ± 0.21	18.55 ± 0.07 76.81 ± 2.33	3.34 ± 0.03 41.05 ± 1.10	3.12 ± 0.04 34.29 ± 2.51
Xylitol (g/L)	Day 8 Day 14*	0.29 ± 0.02 0.94 ± 0.05	0.48 ± 0.04 1.0 ± 0.04	0.27 ± 0.00 0.50 ± 0.01	0.63 ± 0.04 4.06 ± 0.06	1.26 ± 0.04 12.14 ± 0.8
Xylose consumption (g/L/h)	Day 8 Day 14*	0.02 0.09	0.02 0.11	0.005 0.04	0.08 0.14	0.09 0.16
Xylose consumption rate (g/g CDW/h)	Day 8 Day 14*	0.02 0.07	0.03 0.13	0.01 0.05	0.05 0.09	0.04 0.05
Xylitol yield (g/g) of consumed xylose	Day 8 Day 14*	0.08 0.03	0.10 0.03	0.14 0.05	0.04 0.09	0.08 0.23
Percentage change in CDW improvement	Day 8 Day 14*	– –	− 3.67 − 10.78	− 46.17 − 32.31	+ 68.48 + 13.00	+ 120.81 + 114.90
Maximum Percentage change in xylitol yield	Day 8 Day 14*	– –	+ 68.42 + 6.95	− 5.26 − 46.81	+ 119.30 + 334.22	+ 340.35 + 1198.90

Results of at least two independent experiments performed in triplicates were shown

**Fig. 4:**
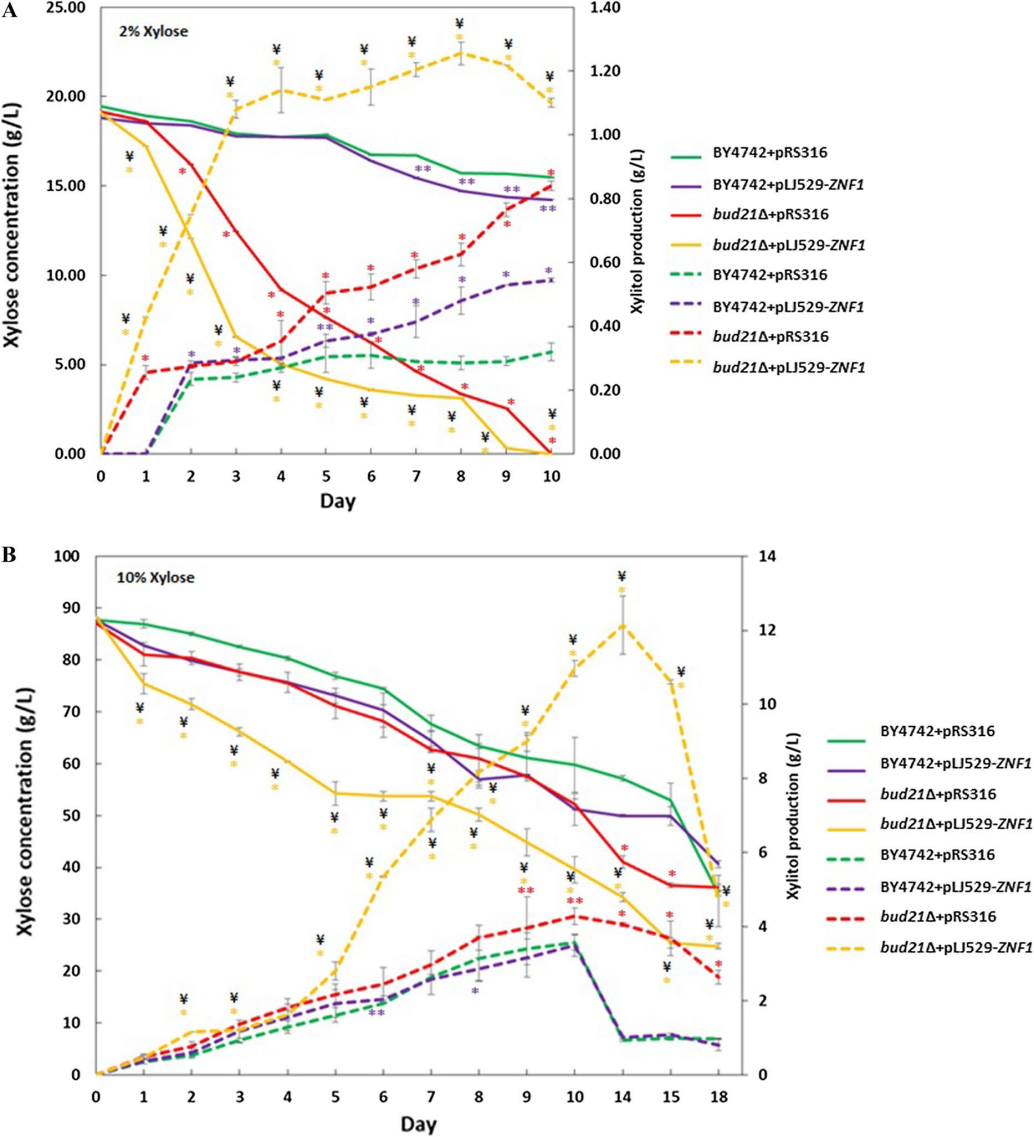
Xylose fermentation profile and xylitol production of *S. cerevisiae* wild type and engineered strains. Different *S. cerevisiae* strains of BY4742 + pRS316, BY4742 + pLJ529-*ZNF1*, *bud21*∆ + pRS316, and *bud21*∆ + pLJ529-*ZNF1* were grown under YPX supplemented with **A** 2% (w/v) or **B** 10% (w/v) of xylose mixed with 0.05% glucose at 30 °C for 10 or 18 days, respectively. For all plots presented, xylose concentration (g/L) (solid line), xylitol concentration (g/L) (dashed line). Xylose consumption and xylitol concentrations were determined by HPLC, and data was based on two independent experiments conducted in triplicate. Error bars indicated standard deviations calculated from at least two independent experiments performed in triplicate. Significance differences were determined by one-way ANOVA with Tukey HSD method (*, p < 0.05; **, p < 0.01, as compared to the control BY4742 + pRS316 strain or ¥, p < 0.05 as compared to the control *bud21*∆ + pRS316)

Under 2% (w/v) xylose and 0.05% (w/v) glucose mix conditions, xylose was completely used by day 10 of fermentation (Fig. 4A). Xylose consumption was highest in the *bud21*∆ + pLJ529-*ZNF1* strain at 0.09 g/L/h or approximately at a rate of 0.04 g/g CDW/h when compared to BY4742 + pRS316 (Table 1). Since Znf1 not only activated xylose reductase genes but also xylitol dehydrogenase genes (Additional file 1: Table S1 and Fig. 2D), xylitol was rapidly consumed by the engineered strain during xylose starvation (Fig. 4A). In the metabolic profiling of the *znf1*∆ + pRS316 strain, carrying a deletion of the *ZNF1* gene, it showed a decrease in xylitol yield by 5.26%, or at 0.27 g/L of maximum xylitol produced, compared with the wild-type strain with a yield of 0.29 g/L of maximum xylitol produced from 2% xylose fermentation at day 8 (Table 1). However, overexpression of *ZNF1* significantly increased the xylitol yield up to 68.42% or 0.48 g/L xylitol when compared to the BY4742 + pRS316 strain with a yield of 0.29 g/L xylitol (Table 1). The *bud21*∆ + pRS316 strain, carrying a deletion of the *BUD21* gene, showed a greater increase in xylitol yield of 119.30%, or 0.63 g/L of maximum xylitol produced at day 8 when compared to the wild-type strain (Table 1). Interestingly, the *bud21*∆ + pLJ529-*ZNF1* strain consumed xylose much faster than the *bud21*∆ + pRS316 strain, with a significantly increased xylitol yield of 340.35%, i.e. 1.26 g/L xylitol or 0.08 g of xylitol/g of consumed xylose (Table 1) or approximately 6-times higher than the wild-type stain. Overall, the results indicate that this engineering approach of *ZNF1* overexpression and *BUD21* deletion is promising for enhanced xylose fermentation.

Next, a higher concentration of xylose was used to augment xylitol production using a 0.05% (w/v) glucose and 10% (w/v) xylose mix. At this xylose concentration, over 60% of xylose was used by the wild-type *S. cerevisiae* at the end of fermentation on day 18 (Fig. 4B). Similarly, at day 14, the *znf1*∆ + pRS316 strain showed a decrease in xylitol yield by 46.81%, or 0.50 g/L of maximum xylitol produced, compared with the wild-type strain with a yield of 0.94 g/L of maximum xylitol produced (Table 1). Overexpression of *ZNF1* significantly increased the xylitol yield by 6.95%, or 1.00 g/L xylitol and consumed xylose at 0.11 g/L/h, comparable to the wild-type strain (Fig. 4B and Table 1). The *bud21*∆ + pRS316 strain showed an increase in xylitol yield by 334.22%, or 4.06 g/L of maximum xylitol produced, which is 4.0-fold higher than the wild-type strain at day 14 (Table 1). Interestingly, the *bud21*∆ + pLJ529-*ZNF1* strain consumed xylose much faster than the other strains at a rate of 0.16 g/L/h, with a significantly increased xylitol yield of 1,198.90%, or 12.14 g/L xylitol (Table 1), i.e. 12-fold higher than the wild-type strain, supporting the current engineering approach to high xylose to xylitol bioconversion. Notably, during days 10 to 14 of fermentation, xylitol production declined, suggesting the consumption of xylitol as another source of carbon (Fig. 4B). This was observed for all tested strains, especially those overexpressing Znf1, suggesting its role in the conversion of xylitol to xylulose and other downstream metabolites.

Additionally, regarding cell growth, the *znf1*∆ + pRS316 strain displayed a significant decrease in biomass by 32.31–46.17% and a biomass specific rate of 0.003 g/L/h while the overexpressing BY4742 + pLJ529-*ZNF1* strain showed lower cell growth by 3.67–10.78% and biomass specific rates of 0.003–0.005 g/L/h when compared to the BY4142 + pRS316 strain with biomass specific rates of 0.004–0.005 g/L/h (Table 1). However, the *bud21*∆ + pRS316 strain, which lacks the ribosomal subunit Bud21, displayed a significant increase in biomass by 13–68.48% with biomass specific rates of 0.004–0.009 g/L/h. Further, biomass increased by 114.90–120.81% and higher biomass specific rates of 0.009–0.011 g/L/h were found in the *bud21*∆ + pLJ529-*ZNF1* strain (Table 1). Overall, these results indicate that the transcription factor Znf1 and the xylose suppressor Bud21 affect cell growth and biomass generation during fermentation in xylose.

### Comparative proteome analysis of engineered *S. cerevisiae* strains during xylose utilisation

Proteomic profiles of some selected *S. cerevisiae* strains, namely BY4742, BY4742 + pLJ529-*ZNF1*, and *bud21*∆ + pLJ529-*ZNF1* during growth in YP media containing 0.05% glucose (w/v) mixed with or without 2% xylose (w/v) were obtained at 48 h of fermentation at the highest rate of xylose consumption and no glucose to investigate differential changes in protein expression. A total of 267 differentially expressed proteins were identified, as listed in Tables 2 and 3. The relative quantity was indicated in term of protein fold-change significantly at the 95% confidence level (*P* < 0.05) in triplicate samples. During xylose utilisation, alterations in the protein profile were observed for many genes involved in xylose metabolism, the PPP, hexose transport, and other related pathways (Tables 2 and 3). For example, a large number of proteins related to ribosomal proteins and translation, including the ribosomal 40S subunit (Rps10a, Rps10b, Rps12, Rps13, Rps15, Rps18a, Rps18b, Rsp22a, Rps22b, Rps25a, Rps25b, Rps3, Rps4a, Rps4b), the ribosomal 60S subunit (Rpl1a, Rpl1b, Rpl20a, Rpl20b, Rpl23a, Rpl30, Rpl31a, Rpl32, Rpl4a, Rpl4b, Rpl6a, Rpl9a, and Rpl9b), the ubiquitin-ribosomal 40S subunit (Rps31), the ubiquitin-ribosomal 60S subunit (Rpl40a, and Rpl40b), and translation elongation factor (Eft1, Gcd11, Efb1, Cam1, Tef1, Yef3, Tef4, and Tif1) were affected as their levels of protein expression were differentially increased (> twofold) in *bud21*∆ + pLJ529-*ZNF1* (Table 3). These proteomic results indicate that xylose metabolism is tightly associated with ribosomal protein synthesis. *ZNF1* and *BUD21* appear to play a key role in finetuning protein synthesis during the utilisation of alternative sugars. Recently, the overexpression of *RPL9A, RPL7B,* and *RPL7A* was shown to increase the specific xylose utilisation rate by 6–21% [29], and they were also regulated by these two modulators. In addition, expression levels of many enzymes involved in glycolysis were altered by xylose and depended greatly on Znf1 and Bud21. These were key metabolic enzymes of glycolysis, including Pfk1, Pfk2, Glk1, Tdh3, Hxk1, Hxk2, Pgk1, Eno1, Eno2, Pdb1, Pyk1, Pdc1, Pdc5, and Pdc6, and TCA enzymes such as Aco1, Cit1, and Lat1, which were suppressed by the presence of Znf1 despite the absence of glucose, as shown by low protein levels in the *ZNF1*-overexpressing strain. In contrast, increased protein levels of some gluconeogenic enzymes such as Tdh1/2 was also observed in the *ZNF1*-overexpressing strain, indicating a tight regulation of carbon source utilisation in the absence of glucose (Table 2). However, these protein levels were increased following *BUD21* deletion during xylose utilisation after the post-glucose effect (Tables 2 and 3). In support of this, utilisation of xylose requires glucose-6-phosphate generated from gluconeogenesis and NADPH from the oxidation of G6P via the PPP in response to xylose [48], which was enhanced in the strain lacking the Bud21 suppressor of xylose-utilising proteins (Table 2). Deletion of *BUD21* was also required for increased levels of alcohol dehydrogenase Adh1 during alcoholic fermentation and Gnd1, Tal1, and Tkl1 of the PPP as well as a less known aldo–keto reductase Ydl124w that is involved in the bioconversion of xylose to xylitol (Table 2), supporting the critical role of Bud21 in xylose utilisation.

**Table 2:** Proteomic profiles of central carbon metabolism of *S. cerevisiae* strains BY4742, BY4742 + pLJ529-*ZNF1*, and the *bud21*∆ + pLJ529− *ZNF1* during fermentation of mixed 0.05% glucose (w/v) with or without 2% xylose (w/v) at 48 h

Description/strain		Protein fold-change in xylose versus no xylose condition			
Protein	Function	BY4742	BY4742	*bud21*∆	*p*-value
			+ pLJ529-*ZNF1*	+ pLJ529-*ZNF1*	
*Glycolysis/Gluconeogenesis/TCA cycle*					
Pfk1	6-phosphofructokinase 1 and 2	0.91–0.96	0.34–0.59	1.95–3.50	< 0.05
Fba1	Fructose-bisphosphate aldolase	1.04	0.86	1.69	< 0.05
Glk1	Glucokinase	0.89	0.51	4.85	< 0.05
Pgi1	Glucose-6-phosphate isomerase	1.39	1.05	1.91	< 0.05
Tdh1-3	Glyceraldehyde-3-phosphate dehydrogenase 1–3	0.87–0.88	0.74–1.34	1.23–1.90	< 0.05
Hxk1, 2	Hexokinase 1 and 2	1.04–1.29	0.20–0.29	4.01–8.02	< 0.05
Pgk1	Phosphoglycerate kinase	1.11	0.55	2.51	< 0.05
Gpm1	Phosphoglycerate mutase	0.93	1.1	1.39	< 0.05
Eno1, 2	Enolase 1 and 2	0.93–0.94	0.3	2.57–2.73	< 0.05
Pdb1	Pyruvate dehydrogenase	1.28	0.98	4.25	< 0.05
Pyk1	Pyruvate kinase	0.89	0.57	2.42	< 0.05
Tpi1	Triose-phosphate isomerase	1.28	0.72	2.02	< 0.05
Pdc1, 5, 6	Pyruvate decarboxylase 1, 5, and 6	0.66–0.79	0.37–0.52	1.74–3.37	< 0.05
Aco1	Aconitate hydratase	0.98	0.66	2.92	< 0.05
Cit1	Citrate synthase	0.81	0.56	3.27	< 0.05
Lat1	Dihydrolipoyllysine-residue acetyltransferase	0.76	0.8	3.09	< 0.05
Mdh1	Malate dehydrogenase	1.33	1.05	1.86	< 0.05
Pck1	Phosphoenolpyruvate carboxykinase	0.33	0.5	1.18	< 0.05
*Pentose phosphate pathway and Oxidoreductase*					
Adh1, 2	Alcohol dehydrogenase 1 and 2	0.74–0.98	0.90–1.16	1.05–2.45	< 0.05
Gnd1	Phosphogluconate dehydrogenase	0.95	0.77	2.67	< 0.05
YDL124W	Aldo–keto reductase	0.71	0.3	3.73	< 0.05
Tal1	Transaldolase	0.93	0.4	2.11	< 0.05
Tkl1	Transketolase	1.37	0.87	2.04	< 0.05
*Hexose transport*					
Hxt1-7	Hexose transporter	1.36–1.76	0.57–0.82	3.49–10.43	< 0.05

**Table 3:** Proteomic profiles of ribosomal proteins and translation of *S. cerevisiae* strains BY4742, BY4742 + pLJ529-*ZNF1*, and the *bud21*∆ + pLJ529-*ZNF1* during fermentation of mixed 0.05% glucose (w/v) with or without 2% xylose (w/v) at 48 h

Description/strain		Protein fold-change in xylose versus no xylose condition			
Protein	Function	BY4742	BY4742	*bud21*∆	*p*-value
			+ pLJ529-*ZNF1*	+ pLJ529-*ZNF1*	
*Ribosomal protein and translation*					
Tma19	Protein that associates with ribosomes	0.99	0.65	2.96	< 0.05
Eft1	Elongation factor 2	0.78	0.44	2.2	< 0.05
Gcd11	Translation initiation factor eIF2 subunit gamma	0.78	0.44	2.2	< 0.05
YNL208W	Hypothetical protein interacts with ribosomes	1.24	1.28	1.69	< 0.05
Dug1	Metallodipeptidase	0.86	0.84	2.62	< 0.05
Efb1	Translation elongation factor 1 subunit beta	0.73	0.23	6.02	< 0.05
Cam1	Translation elongation factor EF1B gamma	1.15	0.73	2.64	< 0.05
Arc1	Protein that binds tRNA	1.4	1.07	3.2	< 0.05
Rim1	Single-stranded DNA-binding protein	1.15	1.9	2.06	< 0.05
Pab1	Polyadenylate-binding protein	0.94	0.98	1.51	< 0.05
Rps0-30	Ribosomal 40S subunit protein A and B	0.24–2.04	0.37–1.42	0.40–4.70	< 0.05
Rpl1-43	Ribosomal 60S subunit protein A and B	0.45–1.33	0.44–2.02	0.56–2.69	< 0.05
Rpp0-1	Ribosomal protein P0 and P1B	1.09	1.11	2.83–5.08	< 0.05
Tef1	Translation elongation factor EF-1 alpha	1.05	0.89	1.75	< 0.05
Yef3	Translation elongation factor EF-3	1.05	0.89	2.66	< 0.05
Tef4	Translation elongation factor EF1B gamma	0.97	0.73	2.6	< 0.05
Tif1	Translation initiation factor eIF4A	0.97	0.73	2.37	< 0.05
Rps31	Ubiquitin-ribosomal 40S subunit	1.11	0.97	1.72	< 0.05
Rpl40	Ubiquitin-ribosomal 60S subunit A and B	1.11	0.97	2.61	< 0.05

Moreover, the levels of hexose transporters (Hxt1, 2, 3, 5, 6, and 7) were decreased in the *ZNF1*-overexpressing strain as compared to the wild-type strain BY4742 (Table 2), suggesting a repressive role of Znf1 on the transport of glucose during co-fermentation in the xylose utilising step. Interestingly, dramatically increased protein levels (by approximately tenfold) were observed in the *bud21*∆ + pLJ529-*ZNF1* strain for most Hxts, including Hxt2 and Hxt7, which are required for xylose uptake (Table 2).

### Improved tolerance to lignocellulosic inhibitors in the engineered *bud21*∆ + pLJ529-*ZNF1* strain

**Fig. 5:**
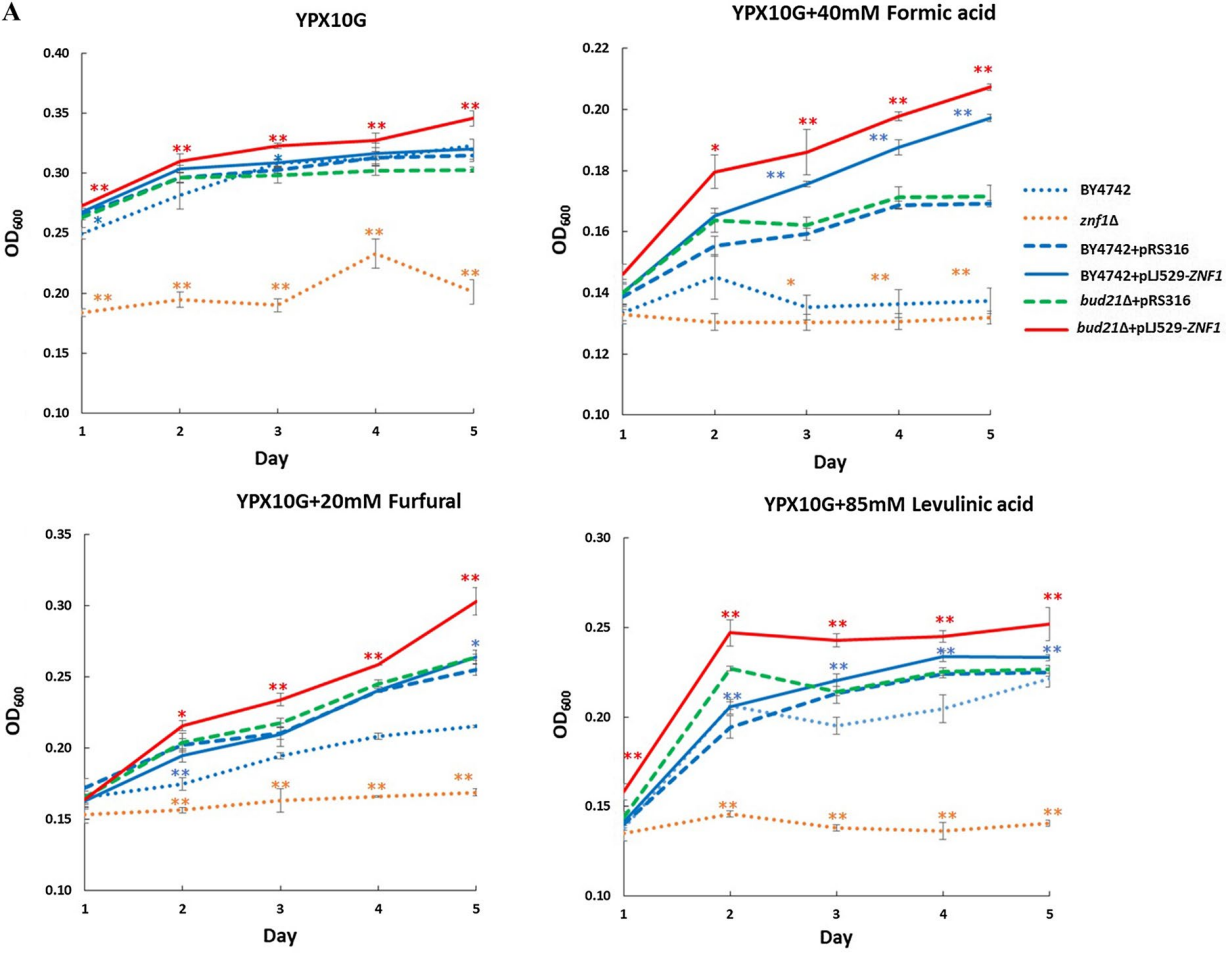

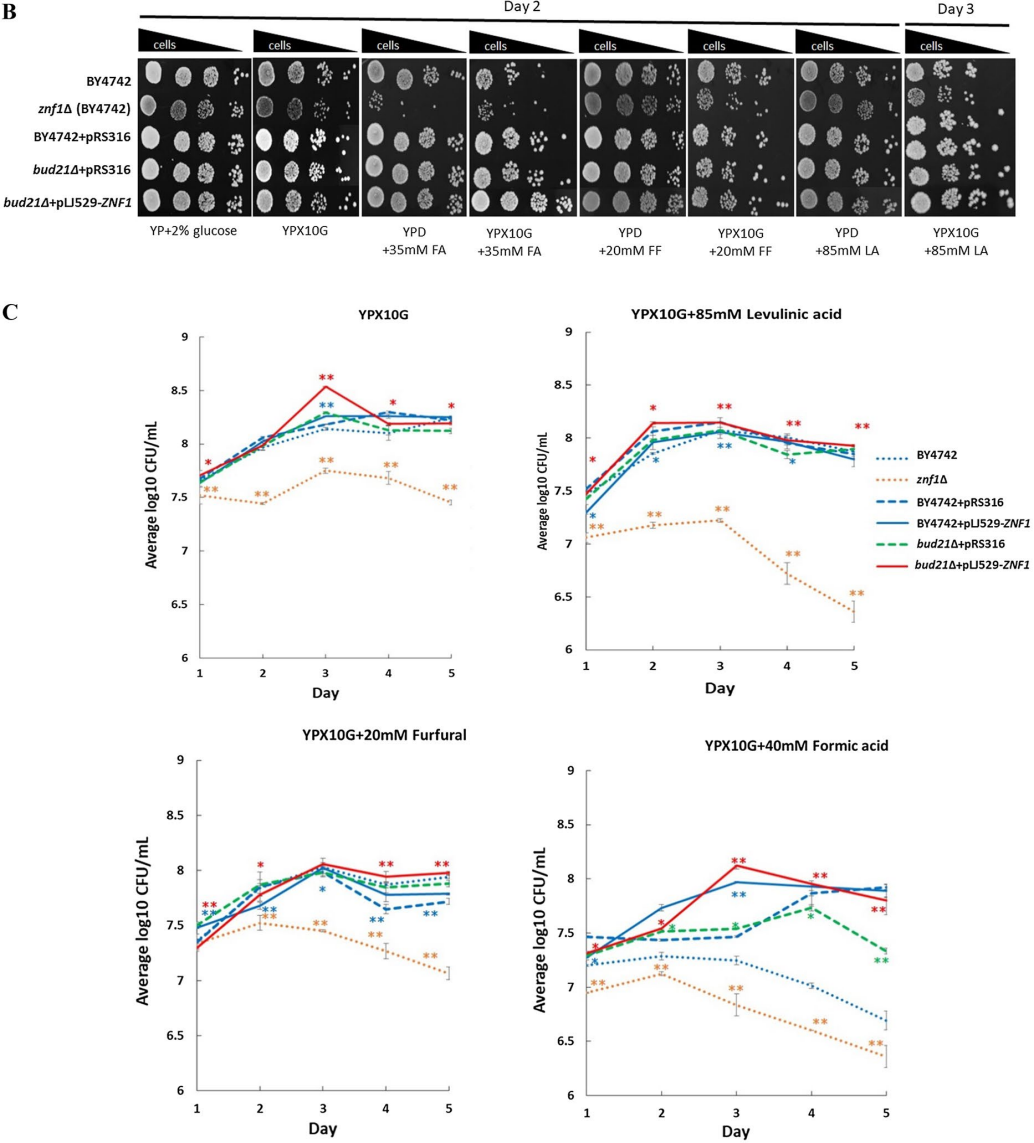
Overexpression of *ZNF1* and deletion of *BUD21* genes conferred tolerance to furfural and lignocellulosic acids stress. The *S. cerevisiae* wild-type BY4742, the *znf1*∆, the BY4742 + pRS316, the BY4742 + pLJ529-*ZNF1*, the *bud21*∆ + pRS316 and *bud21*∆ + pLJ529-*ZNF1* strains were examined for growth and cell survival. **A** Growth assays were conducted. Cells were grown in YPX10 media containing 10% xylose (w/v) and 0.05% glucose plus 20 mM furfural (FF), 40 mM formic acid (FA), or 85 mM levulinic acid (LA). Growth of strains were monitored and expressed as the optical density values (OD_600_) for 5 days at 30 °C. **B** Spot tests of different *S. cerevisiae* strains were examined on YPX10 plates containing10% xylose (w/v) and 0.05% glucose to monitor cell survival in the presence of 35 mM formic acid, 20 mM furfural, or 85 mM levulinic acid. Ten-fold serial dilutions of cells were spotted on plates and incubated at 30 °C for 2–3 days. **C** Cell survival was analyzed using CFU/ml method. Significance differences were determined by one-way ANOVA with Tukey HSD method (*, p < 0.05; **, p < 0.01) as compared to the controls BY4742 or BY4742 + pRS316. Error bars indicated standard deviation (SD)

Xylitol production is also affected by intracellular pools of NADPH and NADH as well as the presence of inhibitory compounds produced during chemical hydrolysis or pretreatment. An additional detoxification step is normally required unless inhibitor tolerant yeast strains are employed. Thus, the involvement of the Znf1 transcription factor in mediating tolerance to lignocellulosic inhibitors was investigated. Deletion of the *ZNF1* gene was found to dramatically impair the growth of yeast cells on 10% xylose-containing YPD plates with the addition of lignocellulose inhibitors such as 40 mM formic acid (FA), 20 mM furfural (FF), or 85 mM levulinic acid (LA) (Fig. 5A). However, the *S. cerevisiae ZNF1*-overexpressing strain displayed better growth as compared to the wild-type strain in the presence of FA and LA (Fig. 5A). Deletion of the xylose suppressor in the *bud21*Δ strain did not significantly improve cell growth, while the *bud21*∆ + pLJ529-*ZNF1* strain showed maximal growth as compared the other strains (Fig. 5A). To confirm, spot assays were also performed in the presence of lignocellulose inhibitors. The most severe impairment was found in the *znf1*Δ, wild-type, *bud21*∆ and *bud21*∆ + pLJ529-*ZNF1* strains, respectively (Fig. 5B).

Furthermore, CFU assays were conducted to assess cell survival, which may not obviously be observed by spot tests. At day 3 of incubation under high xylose conditions (top right panel), the *znf1*∆ + pRS316 strain showed dramatically decreased cell growth and fewer colony-forming units (CFU/mL) of 7.75 × 10^6^ as compared to the wild-type strain with a CFU of 8.18 × 10^6^ (Fig. 5C)_._ Additionally, the *bud21*∆ + pLJ529-*ZNF1* strain displayed a significant increase in CFU to 8.53 × 10^6^, while the control strain BY4742 + pRS316, the *bud21*∆ + pRS316 strain, and the BY4742 + pLJ529-*ZNF1* strain had CFU values of 8.18, 8.29 × 10^6^, and 8.29 × 10^6^, respectively (Fig. 5C). With regard to the effect of the inhibitors, the formation of acids and furan aldehydes resulted in decreased sugar yields. In the presence of 40 mM FA, the *bud21*∆ + pLJ529-*ZNF1* strain displayed a significant increase in CFU to 8.14 × 10^6^ as compared to the other strains (Fig. 5C). Finally, in 20 mM FF treated cells at day 4 of incubation, the *znf1*∆ + pRS316 strain showed dramatically decreased cell growth and fewer colony-forming units (CFU) at 7.26 × 10^6^, while the *bud21*∆ + pLJ529-*ZNF1* strain displayed a significant increase in CFU to 7.94 × 10^6^ as compared to the other strains (Fig. 5C). Overall, these results indicate the increased cell viability of the *bud21*∆ + pLJ529-*ZNF1* strain as shown by significantly increased growth, cell survival, and CFU numbers. Thus, the engineered strain may be applicable for xylose fermentation using acid pretreatment.

### Conversion of xylose to xylitol from rice straw hydrolysate

**Fig. 6:**
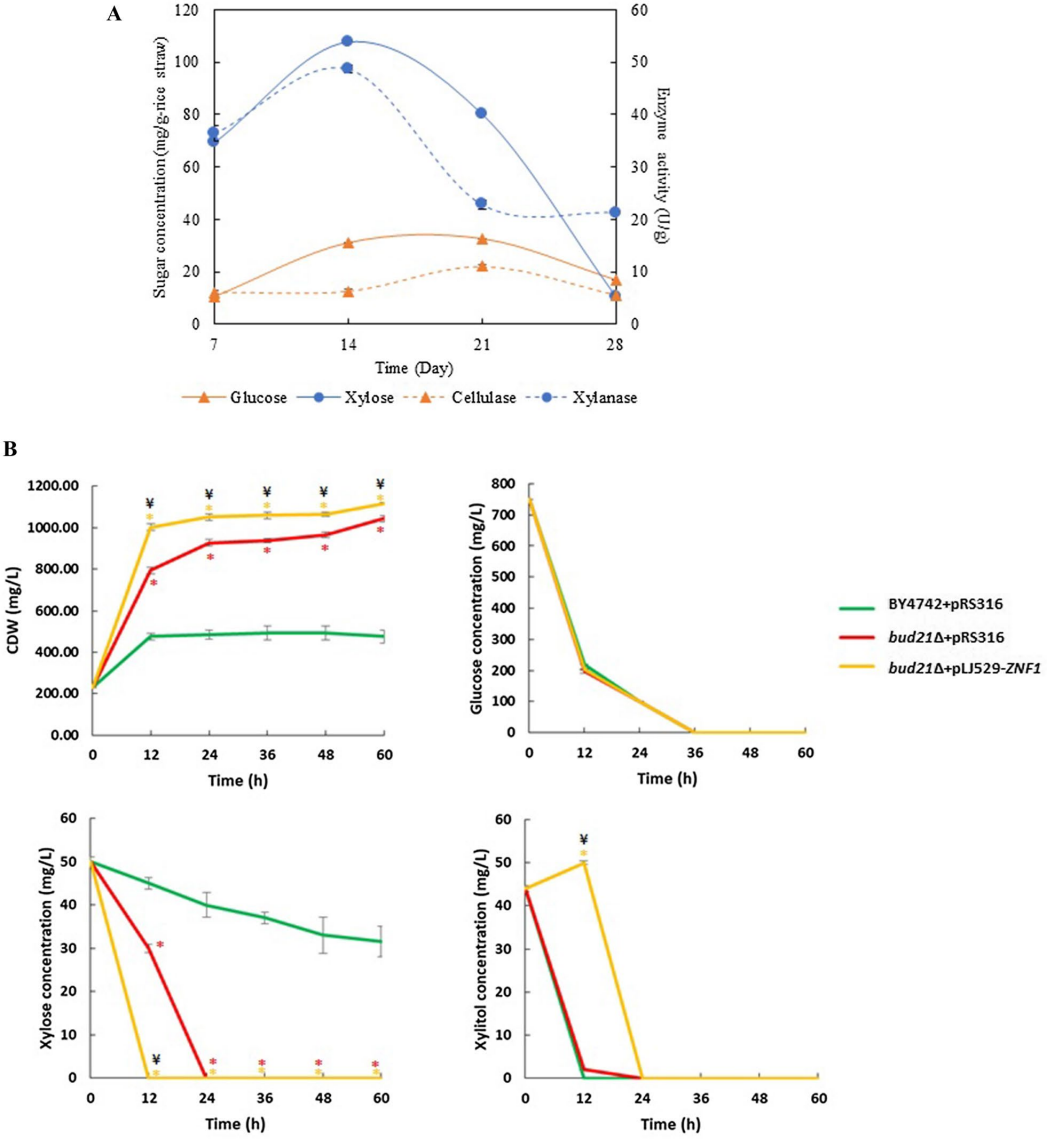
Solid state fermentation of rice straw pretreated with *Xylaria* sp. BCC1067 for xylose to xylitol conversion. **A** Enzyme activity of cellulase and xylanase (U/g) and sugar concentration (mg/g of rice straw) under solid-state fermentation. Rice straw was pretreated with fungi *Xylaria* sp. BCC1067 cultivated for 28 days at 25 °C at 70% moisture content for 28 days. The reaction mixture was incubated at 50 °C for 10 min. The reducing sugars released were quantified using glucose or xylose as a standard. **B** Conversion of xylose to xylitol from rice straw hydrolysate. The BY4742 + pRS316, the BY4742 + pLJ529-*ZNF1*, the *bud21*∆ + pRS316, and the *bud21*∆ + pLJ529-*ZNF1 S. cerevisiae* strains were grown using rice straw hydrolysate supplemented with YP medium and 0.05% glucose. Strains were incubated at 30 °C with shaking for 60 h. Glucose, xylose and xylitol concentrations were determined by HPLC and CDW (mg/L) was also obtained. Error bars indicated standard deviations calculated from at least two independent experiments performed in triplicate. Significance differences were determined by one-way ANOVA with Tukey HSD method (*, p < 0.05; **, p < 0.01) or (¥, p < 0.05) as compared to BY4742 + pRS316 or *bud21*∆ + pRS316, respectively

Here, as proof of principle, xylitol was produced using rice straw hydrolysate as a cheap and abundant agricultural waste. The strategy was to combine bioconversion with a pretreatment step using the engineered yeast strains with an enhanced ability to utilise relevant xylose substrates. First, the fungus *Xylaria* sp. BCC 1067, a wood-decaying fungus, was cultivated under solid state fermentation (SoSF) with 70% moisture for 28 days as the biological pretreatment step with a low-cost and eco-friendly approach. The highest xylanase activity of 48.66 ± 0.73 U/g of rice straw was obtained at day 14, which rapidly decreased to 22.80 ± 0.91 U/g of rice straw after 28 days (Fig. 6A). The results of xylanase production are shown after 14 days of cultivation (Fig. 6A). Likewise, cellulase production between days 7 and 14 of fermentation was 6.02 ± 0.87 to 6.19 ± 0.68 U/g of rice straw (Fig. 6A). The maximum cellulase production was observed after 21 days at 11.04 ± 0.06 U/g of rice straw (Fig. 6A). Almost all enzyme production was decreased after 14 and 21 days, respectively, which may be due to a reduction in the hemicellulose content, followed by the cellulose content. The fungus *Xylaria* produced more xylanase than cellulase (Fig. 6A). The maximum xylose concentration of 107.7 ± 5.35 mg/g of rice straw was obtained after 14 days of fermentation (Fig. 6A). The highest glucose concentrations of 31.10 and 32.63 mg/g of rice straw were detected after 14 and 21 days, respectively (Fig. 6A).

To start fermentation, yeast cells were provided with an additional 250 mg/L of glucose to obtain an initial glucose concentration of 750.0 mg/L, while the xylose and xylitol concentrations were started at 50.0 and 44.0 mg/L, respectively. Here, all tested strains consumed glucose similarly within 36 h (Fig. 6B), indicating that glucose was the first carbon source consumed, and followed by the activation of xylose metabolism. The combined *bud21*∆ + pLJ529-*ZNF1* strain completely consumed xylose within the first 12 h, much faster than the control BY4742 + pRS316 strain, in which only 30% of xylose was consumed after 60 h (Fig. 6B). Implementation of the engineered strain *bud21*∆ + pLJ529-*ZNF1* allowed for the production of xylitol. This was found to be 50 mg/L or 0.12 g/g of *Xylaria*-pretreated rice straw hydrolysate with supplemented nutrients at 12 h, while no xylitol production was observed with other strains (Fig. 6B). Nevertheless, all strains consumed xylitol within 12–24 h. Additionally, the *bud21*∆ + pRS316 strain displayed a significant increase in biomass of 219.12% or 1.04 g/L when compared to the wild-type strain, while *bud21*∆ + pLJ529-*ZNF1* displayed a significant increase in biomass of 234.87% or 1.12 g/L as compared to the control strain at 60 h (Fig. 6B). Overall, these results confirm the role of the transcription factor Znf1 and the xylose suppressor Bud21 in xylose utilisation and xylitol production.

## Discussion

A better understanding of the transcriptional control of xylose-induced metabolic reprogramming in yeasts will allow for the optimisation of yeast-based bioprocesses to produce biofuels and chemicals using the most abundant sugars available on earth, glucose and xylose. In this study, we characterised the roles of the key transcription factor Znf1 in xylose metabolism by *S. cerevisiae*, and potential use of xylose as a carbon source to produce xylitol as an example. It appears that the expression of genes and proteins involved in xylose utilisation, glucose repression, and ribosomal protein synthesis is mediated by the common transcription regulator Znf1 [26, 28] (Tables 2 and 3). The first two processes are regulated in a concerted fashion to allow for appropriate utilisation of available alternative carbon sources for environmental stress adaptation, and largely depend on the transcription factor Znf1 and its key target protein Bud21. Investigation of xylose utilisation by *S. cerevisiae* and other yeasts indicates a global rewiring of metabolic networks with distinct transcriptional and metabolic patterns to glucose-mediated repression [49].

Importantly, the overexpression *ZNF1* or deletion of *BUD21* has a significantly positive effect on xylose utilisation and xylitol production. Previously, using YE media containing 20 g/L xylose*, S. cerevisiae* strain YKB2680, carrying the plasmid p*XYLA* and *XKS1* encoding XI and xylulokinase, produced approximately 0.58 g/L xylitol (0.029 g/g xylose consumed) while the parental strain showed no xylitol production [22]. Recently, the *S. cerevisiae* strain Y-50463 that contains a synthesised yeast codon optimised for the XI gene *YXI* and a plasmid carrying a set of heterologous xylose utilisation genes of *S. stipitis* produced approximately 6 g/L xylitol (0.24 g/g xylose consumed) in mixed glucose and 25 g xylose fermentation under aerobic conditions [43]. Notably, in our study, the *bud21*∆ + pLJ529-*ZNF1* strain produced a higher yield of xylitol at 12.14 g/L using mixed glucose and 100 g xylose (0.23 g/g xylose consumed) when compared to other engineered strains of *S. cerevisiae*, although less than the native xylose assimilating strains with 0.25–0.6 g/g xylose consumed; therefore, additional improvements such as engineering strategies or optimised fermentation processes are required.

In support, the wild-type *S. cerevisiae* strain Y133 was shown to have significantly increased mRNA levels of *ZNF1* and *BUD21* during anaerobic xylose growth from RNA-seq data [33]. Regarding to the role of Bud21 in xylose to xylitol bioconversion, little is known regarding the involvement of this poorly described protein. Bud21 is involved in ribosomal protein biogenesis and processing, as well as responding to oxidative stress conditions [50]. This may explain its contribution to the enhanced biomass of cells. In addition, Bud21 also inhibits xylose fermentation [22]. A previous study reported that deletion of *BUD21* strongly increases the level of Ty1 RNA by 33-fold and positively affects xylose utilisation [51]. Moreover, Bud21 phosphorylates Ira2, encoding GTPase-activating proteins (GAPs) that negatively regulate the Ras pathway or act as inhibitors of the Ras pathway, leading to an increase in the anaerobic specific xylose consumption rate [52]. Accordingly, the *ira2*∆ strain also showed increased sugar uptake rate during growth at a high xylose concentration of 50 g/L [53]. Thus, it appears that Bud21 may function to suppress xylose utilisation. Importantly, the proteomic profiles of strains with *bud21* deletion strongly enhanced the protein levels of many ribosomal protein and translation elongation factors as part of the post-glucose effect (Table 3). Increased expression levels of some ribosomal protein have been shown to enhance xylose utilisation [29]. In addition, the transcription factor Znf1 exerted unexpected roles during xylose utilisation in the absence of glucose by enhancing ribosome synthesis and altering cell metabolism, respectively. Znf1 exhibited low-level glycolysis and a de-repressed the TCA cycle, resembling glucose repression (Tables 2 and 3). Alteration of the metabolic state also affects glucose uptake, as shown by our and other studies. Different approaches to enhance xylose transport, including co-fermentation of xylose and other carbon sources, have been used to increase the utilisation of xylose [45]. In this study, co-fermentation with low glucose efficiently drove expression of the xylose transporters Hxt4, Hxt7, and Gal2 at the transcriptomic (Table 2 and Fig. 2) and the proteomic levels in the post-glucose effect (Table 3). Overall, overexpression of the transcription factor *ZNF1* enhanced xylose utilisation, leading to induced expression of hexose/xylose transporters, altered hexokinase regulation as observed for Hxk1, and increased Znf1 target activation in various pathways mentioned above (Fig. 2D). Even though overexpression of *ZNF1* in combination with deletion of the xylose suppressor *BUD21* allowed for the activation of xylose metabolism and conferred a stress-tolerant improvement regarding lignocellulosic inhibitors, xylose uptake could be further improved, as shown by the proteomic data (Tables 2 and 3). It appears that glucose-sensing systems respond quite profoundly to alterations in the carbon source, including sugars. Additionally, inhibition of the downstream xylitol dehydrogenase enzymes for xylitol to xylulose conversion or xylulose kinase synthase (Xks1) (Additional file 1: Table S1, Figs. 1 and 2) may be necessary. In fact, the *XKS1*-deleted CK17∆*XKS1 S. cerevisiae* strain shows a good capacity for the co-production of xylitol and ethanol using pretreated corn stover slurry [54]. Since most superior xylitol‑producing natural strains are unsafe for use in the food and pharmaceutical industries, this engineered *S. cerevisiae* is promising for future xylitol production. It could be an alternative to the chemical route, which is associated with high costs and environmental damage [55]. To this end, this work will help in maintaining highly efficient xylose metabolism during glucose–xylose co-fermentation, which could be applied for the production of lignocellulosic bioethanol or other high-value bio-based fuels and biochemicals.

## Conclusion

Znf1 is a key transcription factor that positively activates genes in various pathways of xylose metabolism during the transition from glucose to xylose and negatively inhibits the protein synthesis of some glycolytic and TCA enzymes as well as hexose transporters during xylose fermentation in the post-glucose effect to maintain glucose repression. Its main targets include genes in the PPP, gluconeogenesis, glycolysis, gluconeogenesis as well as the TCA cycle and respiration required for oxidative metabolism, metabolite production, energy generation as well as ribosomal protein synthesis and translation in *S. cerevisiae*. Importantly, Znf1 also represses *BUD21*, whose expression is highly critical for xylose utilisation. A remarkable enhancement of xylitol production from xylose by the combination of *ZNF1* overexpression and *BUD21* deletion was ascribed to the activation of xylose reductases and increased xylose utilisation and blocked function of the xylose suppressor, respectively. A microbiological approach via the transcriptional control of xylose metabolism for further improvements in xylose utilisation from lignocellulosic biomass in yeasts has a promising future in support of the sugar industry and the global bioeconomy.

## Materials and methods

### Strains and culture medium

The *S. cerevisiae* wild-type BY4742, *znf1*Δ, *bud21*Δ, or *ZNF1*-overexpressing strains were used for gene expression analysis, phenotypic analysis or fermentation assays (Additional file 2: Table 2). The *ZNF1*-overexpressing strain (BY4742 + pLJ529-*ZNF1*) was constructed using the empty plasmid pRS316 to create pLJ529 + *ZNF1* [56]. The plasmid pRS316 or pLJ529 + *ZNF1* (Table 4) was transformed into different *S. cerevisiae* strains using the LiAc/SS carrier via the PEG method [57]. Colonies were selected on synthetic complete dropout without uracil (SC-uracil) (Sigma) plates supplemented with 20 g/L glucose as the carbon source. The *ZNF1*-overexpressing strain (*ZNF1*-OE) used in this study (Additional file 2: Table 2) was constructed using the CRISPR/Cas9 gene editing technique. *ZNF1* insertion was at chromosome AD7 and, *ZNF1* expression was driven by the *TEF1* promoter [47]. The yeast culture was regularly maintained in Yeast Peptone Dextrose (YPD) media, containing 10 g/L of yeast extract, 20 g/L of bacto-peptone, and 20 g/L of glucose. Yeast Peptone Xylose (YPX) medium, containing 10 g/L of yeast extract, 20 g/L of bacto-peptone, and 20 g/L of xylose (2% xylose for YPX) or 100 g/L of xylose (10% xylose for YPX10).

### Gene induction and quantitative RT-PCR (RT-qPCR)

For gene expression analysis during the glucose–xylose shift, the *S. cerevisiae* wild-type BY4742, *znf1*Δ, *ZNF1*-overexpressing (BY4742 + pLJ529-*ZNF1*), and *bud21*Δ + pLJ529-*ZNF1* strains were cultured overnight in YPD and then regrown to an approximate OD_600_ of 0.6–0.8. Then, they were transferred to YPX and regrown for about 1 h. For gene expression analysis in a low glucose–xylose mix, the *ZNF1*-OE strain was cultured in YPX containing 0.05% glucose to an approximate OD_600_ of 4.0. For RNA extraction, RNAs were isolated using the phenol: chloroform: isoamyl alcohol (25:24:1, v/v) method and purified with the RNeasy Mini Kit (Qiagen, Hilden, Germany). The cDNA was synthesised from 2 µg of total RNAs according to the qPCRBIO cDNA synthesis kit. RT-qPCR was performed with a CFX Connect Real-Time PCR System (Bio-Rad, CA, USA) and the CFX Connect Real-Time PCR System was used for data analysis. The reaction mixtures contained Luna Universal One-Step RT-qPCR Kit (NEB). The sequences of the primers used for RT-qPCR are listed in Table 4. The relative quantification of each transcript was calculated using the 2^−∆∆Ct^ method [58] using the *ACT1* gene as the internal control. All experiments were performed with at least two independent experiments performed in triplicate.

### Xylose fermentation under low glucose–xylose mix conditions

The *ZNF1*-OE strain was grown in YPX under low glucose conditions at a concentration of 0.05%, 0.04%, 0.03%, 0.02%, 0.01% (w/v), or without glucose to examine its xylose utilising ability. Cells were grown at a temperature of 30 °C with shaking at 150 rpm for 96 h, and the OD_600_ of the cell culture was measured using a spectrophotometer and converted into cell biomass. For xylose fermentation, the BY4742 + pRS316, BY4742 + pLJ529-*ZNF1, bud21*Δ + pRS316, and *bud21*Δ + pLJ529-*ZNF1* strains were used (Table 4). Cells were cultured in 50 mL YNB-Ura broth for 16 h, transferred in 250 mL YPD and incubated overnight at 30 °C with shaking at 150 rpm. The strains containing pRS316 or the pLJ529-*ZNF1* plasmid were pre-inoculated in YNB-Ura to maintain the constructs for selection purposes, and the expression level of *ZNF1* was checked using RT-qPCR.

Total cells were resuspended in 5 mL of distilled water and adjusted to an OD_600_ of 1.0 or cell dry weight (CDW) of 0.23 g/L (approximately 0.3 mL of cell solution) in 50 mL of YP broth containing xylose at 10% or 2% (w/v) mixed with 0.05% (w/v) glucose. Cell samples were harvested daily until 10 or 18 days, respectively, for metabolite analysis via HPLC and OD_600_ measurements. The obtained fermentation samples were analysed to determine the concentrations of xylose and glucose, using HPLC (Shimadzu, Japan) with an Aminex HPX-87H ion-exchange column (300 × 7.8 mm i.d.) (Bio-Rad, Hercules, USA). A mobile phase of 5 mM H_2_SO_4_ was used at a flow rate of 0.6 mL/min and a column temperature of 65 °C.

### Protein extraction and analysis

BY4742 and *znf1*Δ were cultured in 50 mL of YPD broth, while BY4742 + pLJ529-*ZNF1* and *bud21*Δ + pLJ529-*ZNF1* were cultured in 50 mL YNB-Ura broth, then transferred to 250 mL YPD and incubated overnight at 30 °C with shaking at 150 rpm. Total cells were resuspended in 5 mL of distilled water and adjusted to an OD_600_ of 1.0 or cell dry weight (CDW) of 0.23 g/L (approximately 0.3 mL of cell solution) in 50 mL of YP broth containing xylose at 2% (w/v) mixed with 0.05% (w/v) glucose. Cell samples were harvested for 48 h. Yeast samples were lysed in 0.2% sodium dodecyl sulphate (SDS) (Amresco, USA), 20 mM dithiothreitol (DTT) (USB, USA), 100 mM NaCl (Bio Basic, USA), and 50 mM Tris–HCl, pH 8.0 (Bio Basic, USA). Proteins were precipitated using cold acetone solution at a 1:5 ratio (v/v) and resolubilised using 0.25% Rapidgest SF (Waters, USA) in 20 mM ammonium bicarbonate (Sigma Aldrich, Denmark). The protein concentrations of the lysates were determined using a BioRad Protein Assay Kit (BioRad, CN). The protein sample in an amount of 25 μg was treated with 4 mM DTT at 72 °C for 30 min to reduce disulphide bonds, then alkylated using 12 mM iodoacetamide [59] (GE Healthcare, UK) at room temperature in the dark for 30 min and desalted using a Zeba spin desalting column (Thermo Scientific, Sweden) prior to digestion using trypsin (Thermo Scientific, Lithuania) at a 1:50 protein:trypsin ratio (w/w) at 37 °C overnight [60]. The solution was evaporated and reconstituted in 0.1% FA (Sigma Aldrich, Denmark) in LC–MS water (Supelco®, Denmark).

A spectral library of yeast for SWATH-MS analysis, 5 μg of each digested sample were pooled. Then, 1 μg of pooled sample was loaded using nanoLC (Thermo Scientific, Denmark) onto a trap column (300 µm i.d. × 5 mm, packed with 5 µm C18 100 Å PepMap™; Thermo Scientific, Denmark) and desalted with 2% acetonitrile (ACN) (VWR, France) and 0.05% trifluoroacetic acid (TFA) (Sigma Aldrich, Denmark) at 10 µL/min for 3 min. Then, the peptides were separated using an analytical column (75 µm i.d. × 15 cm, packed d with Acclaim PepMap™ C18) (Thermo Scientific, Denmark) at 300 nL/min. The elution was carried out with linear gradient of 3–35% of buffer B in A for 92 min (A: 0.1% FA in water; B: 0.1% FA in 80% ACN). The eluted peptides were analysed in 6600plus TripleTOF (LC–MS/MS) (ABSCIEX, Denmark). The MS acquisition time was set from gradient time zero to 120 min, and the MS1 spectra were collected in the mass range of 400 to 1,500 m/z with 250 ms in “high sensitivity” mode. Further fragmentation of each MS1 spectrum occurred with a maximum of 30 precursors per cycle. Switch criteria used were the following: charge of 2 + to 5 + , 500 cps intensity threshold and dynamic exclusion for 15 s.

SWATH-MS data for individual samples were acquired by LC–MS/MS exactly as described above. SWATH acquisition was carried out in data-independent acquisition (DIA) mode. The MS1 spectra were collected in the mass range of 400 to 1,250 m/z in “high sensitivity” mode. The variable Q1 isolation windows were optimised based on the spectral library using the SWATH Acquisition Variable Window Calculator (https://sciex.com/software-support/software-downloads). Collision energy was different for each window. Single injections of biological triplicates were performed.

The spectral library was processed using ProteinPilot™ Software 5.0.2 (ABSCIEX, De) with the *Saccharomyces cerevisiae* database (UniProtKB). The proteins identified by LC–MS/MS in each pooled yeast sample with an unused score above 0.05 (> 95% confidence) and a false discovery rate (FDR) lower than 1% were considered significant and included in the subsequent analyses. The SWATH-MS data were analysed using PeakView 2.2 software (ABSCIEX, Denmark). The generated spectral library was used as a database for SWATH analysis. Data were processed using an XIC extraction window of 5 min and XIC width of 75 ppm. Peak areas from peptides with > 95% confidence and a < 1% global false discovery rate was extracted using MarkerView v1.3.0 (ABSCIEX, Denmark). All annotations were derived from the *Saccharomyces* Genome Database (SGD) (http://www.yeastgenome.org/). Cluster analysis was performed using DAVID bioinformatics resources (https://david.ncifcrf.gov/home.jsp) [61, 62].

### Growth and inhibitor tolerance assays

*S. cerevisiae* strains were examined for their ability to grow on YPX or YPX-low glucose with or without lignocellulosic inhibitors including FA, LA, and FF. They were grown in YPD broth overnight at 30 °C with shaking at 150 rpm. Yeast cells were harvested and resuspended in distilled water and diluted to an OD_600_ of 0.1, 0.01, 0.001, and 0.0001. Then, cells were spotted onto YPX agar plates containing 0.05% glucose or different xylose concentrations, i.e. 2% or 10% (w/v). Cells were grown at 30 °C for 2–5 days. Yeast cells were inoculated and cultured at an initial OD_600_ of 0.1 using 96-well plates, containing 0.2 mL YPX10 + 0.05% (w/v) glucose with or without 35 mM FA, 85 mM LA, or 20 mM FF. Cells were grown at 30 °C with shaking 150 rpm for 5 days. The samples were collected at every 24 h to measure cell density using a Multiskan Sky Microplate spectrophotometer. For the colony forming unit (CFU) count assay, cell samples were diluted and spread onto YPD plates that were incubated at 30 °C for 48 h.

### Xylitol production from rice straw hydrolysate

#### Rice straw preparation and *Xylaria* sp. BCC1067 culture

Rice straw was collected from a rice field in Chaiyaphum province, Thailand. Rice straw was dried at 60 °C for 2 days, then shredded into 5–10 mm pieces, stored in plastic bags, and kept at room temperature before use. The fungus *Xylaria* sp. BCC 1067 was obtained from the BIOTEC Culture Collection (BCC culture 6,200,032,292; National Science and Technology Development Agency, Bangkok, Thailand). For inoculum preparation, the *Xylaria* culture was inoculated on potato dextrose agar (PDA) plates and incubated at 25 ± 2 °C for 7 days. The base liquid medium contained 10 g/L peptone and 10 g/L yeast extract.

#### Solid-state fermentation

10 g of dried rice straw was placed in a 500 mL Erlenmeyer flask. Small pieces of agar (plug size 2 × 2 mm) cut from actively growing fungal mycelium were used as the inoculum. The initial moisture content was adjusted to 70% with the base medium and samples were incubated at 25 ± 2 °C for 28 days. For time-course studies, whole flask replicates were collected at designated time points. The sample was extracted with 0.05 M acetate buffer pH 5.0 by shaking in a rotary shaker at 150 rpm for 2 h and centrifuged at 8000 rpm for 10 min at 4 °C. The supernatant was used to determine the enzyme activity.

#### Cellulase and xylanase activity

The cellulase activity of the fermented liquid was determined according to [63]. For this, 0.5 mL of enzyme solution was added to 0.5 mL of 1% carboxymethylcellulose (CMC) in 0.05 M citrate buffer (pH 5.0). The reaction mixture was incubated at 50 °C for 10 min. The released reducing sugars were quantified using glucose as a standard. One unit of cellulase (IU) was defined as the amount of enzyme releasing 1 μM of glucose per min under the assay conditions. The xylanase activity of the fermented liquid was determined according to a previous publication [64]. Then, 0.5 mL of enzyme solution was added to 0.5 mL 1% xylan dissolved in 0.05 M citrate buffer (pH 5.0). The reaction mixture was incubated at 50 °C for 10 min. The released reducing sugars were quantified using xylose as the standard. One unit of xylanase was defined as the amount of enzyme releasing 1 μM of xylose equivalents per minute under the assay conditions.

#### Rice straw hydrolysate fermentation

The BY4742 + pRS316, *bud21*Δ + pRS316, and *bud21*Δ + pLJ529-*ZNF1 S. cerevisiae* strains were cultured in YPD broth and incubated overnight at 30 °C with shaking at 150 rpm. Total cells were resuspended in distilled water and diluted to an OD_600_ of 1.0, then transferred into 50 mL of YP broth with liquid rice straw hydrolysate and 0.05% glucose. Cell samples (1.5 mL) were harvested until 60 h for OD_600_ measurements. The fermentation samples were analysed using HPLC as previously described.

## Supplementary Information

**Additional file 1**: Gene targets of Znf1 in the central carbon metabolism during growth on the glucose-xylose shift.

**Additional file 2**: List of plasmids, primers and strains used in this study.
