# Supplementary material for: Activation of cryptic xylose metabolism by a transcriptional activator Znf1 boosts up xylitol production in the engineered Saccharomyces cerevisiae lacking xylose suppressor BUD21 gene
Source: Microb Cell Fact. 2022 Mar 5;21:32. doi: 10.1186/s12934-022-01757-w (PMC8897867; doi:10.1186/s12934-022-01757-w)
Supplement: Supplementary file 1 — Additional file 1: Gene targets of Znf1 in the central carbon metabolism during growth on the glucose-xylose shift. [file 12934_2022_1757_MOESM1_ESM.docx]

**Additional file 1: Table 1 Gene targets of Znf1 in the central carbon metabolism during growth on the glucose-xylose shift.** Gene expression changes in the *S. cerevisiae* the wild-type BY4742 versus deletion *znf1*Δ strains were listed. Strains were cultured in rich YPD media containing 2% glucose until mid-log phase of growth and then transferred to YP media containing 2% xylose and grown for additional 1 h. (see material and method for more details). The average values were calculated from at least two independent experiments performed in three replicates.

| **Localization** | **ORF** | **Gene** | **Function** | **Fold change**  **in WT (Xylose/Glucose)** | **Fold change**  **(WT/*znf1*∆)**  **2% Xylose** | **Fold change**  **(WT/*znf1*∆)**  **2% Glucose** |
| --- | --- | --- | --- | --- | --- | --- |
| **Xylose metabolism** | *YOR120W* | *GCY1* | Glycerol dehydrogenase | 2.8±0.07 | 3.3±0.04 | 0.06±3.68 |
|  | *YHR104W* | *GRE3* | Aldose reductase | 22.3±0.43 | 25.0±0.03 | 0.1±0.00 |
|  | *YDL124W* | *-* | NADPH-dependent alpha-keto amide reductase | 3.1±0.22 | 3.3±0.02 | 1.1±0.05 |
|  | *YJR096W* | *-* | Xylose and arabinose reductase | 2.0±0.12 | 2.5±0.02 | 1.3±0.22 |
|  | *YDR368W* | *YPR1* | NADPH-dependent aldo-keto reductase | 2.4±0.14 | 1.7±0.07 | 0.09±1.33 |
|  | *YLR070C* | *XYL2* | Xylitol dehydrogenase | 3.0±0.45 | 3.1±0.05 | 0.1±0.00 |
|  | *YJR159W* | *SOR1* | Sorbitol dehydrogenase | 2.0±0.36 | 2.0±0.18 | 0.7±0.01 |
|  | *YDL246C* | *SOR2* | Sorbitol dehydrogenase | 2.3±0.35 | 2.0±0.15 | 0.8±0.10 |
|  | *YGR194C* | *XKS1* | Xylulokinase | 2.9±0.60 | 1.1±0.18 | 0.1±0.00 |
|  | *YOR078W* | *BUD21* | Component of small ribosomal subunit/ xylose suppressor | 2.2±0.05 | 2.0±0.06 | 2.0±0.02 |
| **PPP** | *YNL241C* | *ZWF1* | Glucose-6-phosphate dehydrogenase | 3.1±0.99 | 2.0±0.12 | 2.0±0.02 |
|  | *YHR163W* | *SOL3* | 6-phosphogluconolactonase | 0.4±0.12 | 0.4±0.20 | 1.4±0.48 |
|  | *YGR248W* | *SOL4* | 6-phosphogluconolactonase | 0.4±0.92 | 0.3±0.36 | 0.6±0.10 |
|  | *YHR183W* | *GND1* | 6-phosphogluconate dehydrogenase | 5.5±0.07 | 0.7±0.07 | 0.7±0.66 |
|  | *YOR095C* | *RKI1* | Ribose-5-phosphate ketol-isomerase | 5.9±0.01 | 1.0±0.00 | 10±0.03 |
|  | *YJL121C* | *RPE1* | Ribulose-5-phosphate 3-epimerase | 0.4±0.01 | 0.4±0.53 | 1.3±0.14 |
|  | *YPR074C* | *TKL1* | Transketolase | 4.0±0.01 | 0.6±0.00 | 1.3±0.17 |
|  | *YBR117C* | *TKL2* | Transketolase | 2.2±0.90 | 10.0±0.05 | 1.1±0.10 |
|  | *YLR354C* | *TAL1* | Transaldolase | 2.2±1.05 | 3.3±0.01 | 1.4±0.09 |

| **Localization** | **ORF** | **Gene** | **Function** | **Fold change**  **in WT (Xylose/Glucose)** | **Fold change**  **(WT/*znf1*∆)**  **2% Xylose** | **Fold change**  **(WT/*znf1*∆)**  **2% Glucose** |
| --- | --- | --- | --- | --- | --- | --- |
| **Glycolysis** | *YCL040W* | *GLK1* | Glucokinase | 1.5±0.11 | 2.0±0.39 | 2.5±0.04 |
|  | *YFR053C* | *HXK1* | Hexokinase isoenzyme 1 | 25.6±0.01 | 2.0±0.03 | 0.4±0.02 |
|  | *YGL253W* | *HXK2* | Hexokinase isoenzyme II | 0.4±0.01 | 2.0±0.03 | 0.7±0.03 |
|  | *YBR196C* | *PGI1* | Phosphoglucose isomerase | 0.5±0.08 | 0.9±0.39 | 0.8±0.00 |
|  | *YGR240C* | *PFK1* | Phosphofructokinase I | 1.2±0.12 | 1.0±0.02 | 33.3±0.00 |
|  | *YMR205C* | *PFK2* | Phosphofructokinase II | 2.1±0.01 | 0.1±0.00 | 0.7±0.02 |
|  | *YKL060C* | *FBA1* | Fructose 1,6-bisphosphate aldolase | 1.0±0.55 | 0.8±0.82 | 0.6±0.03 |
|  | *YDR050C* | *TPI1* | Triose phosphate isomerase | 0.5±0.05 | 1.4±0.08 | 1.4±0.06 |
|  | *YJL052W* | *TDH1* | Glyceraldehyde-3-phosphate dehydrogenase | 3.9±0.79 | 0.8±0.43 | 0.3±0.11 |
|  | *YCR012W* | *PGK1* | 3-phosphoglycerate kinase | 1.4±0.00 | 1.3±0.03 | 50.0±0.10 |
|  | *YKL152C* | *GPM1* | Phosphoglycerate mutase | 0.5±0.70 | 1.1±0.07 | 2.5±0.10 |
|  | *YGR254W* | *ENO1* | Enolase | 2.2±0.54 | 2.0±0.14 | 1.4±0.10 |
|  | *YAL038W* | *PYK1* | Pyruvate kinase | 0.9±0.33 | 1.7±0.03 | 0.7±0.10 |
| **Glycerol metabolism** | *YDL022W* | *GPD1* | Glycerol-3-P dehydrogenase | 0.9±0.09 | 0.8±0.04 | 1.4±0.17 |
|  | *YOL059W* | *GPD2* | Glycerol-3-P dehydrogenase | 1.4±0.61 | 0.6±0.65 | 1.1±0.14 |
|  | *YIL053W* | *GPP1*  *(RHR2)* | Glycerol-3-P phosphatase | 1.2±0.02 | 1.3±0.19 | 1.0±0.20 |
|  | *YER062C* | *GPP2*  *(HOR2)* | Glycerol-3-P phosphatase | 0.7±0.12 | 0.8±0.74 | 1.0±0.14 |
| **Ethanol metabolism** | *YGL062W* | *PYC1* | Pyruvate carboxylase | 2.8±0.12 | 1.7±0.36 | 0.2±0.06 |
|  | *YLR044C* | *PDC1* | Pyruvate decarboxylase | 0.7±0.04 | 1.1±0.04 | 2.0±0.10 |
|  | *YOL086C* | *ADH1* | Alcohol dehydrogenase I | 0.5±0.10 | 0.8±0.17 | 3.3±0.09 |
|  | *YMR303C* | *ADH2* | Alcohol dehydrogenase II | 9.1±1.76 | 0.9±0.17 | 0.4±0.09 |
| **TCA cycle /Respiration** | *YKL148C* | *SDH1* | Succinate dehydrogenase | 14.1±0.01 | 2.0±0.00 | 0.2±0.00 |
|  | *Q0045* | *COX1* | Cytochrome c oxidase | 6.4±1.13 | 0.3±0.02 | 100.0±0.00 |
|  | *YAL039C* | *CYC3* | Cytochrome c heme lyase | 3.1±0.57 | 1.3±0.08 | 0.3±0.05 |
|  | *YDL215C* | *GDH2* | Glutamate dehydrogenase | 7.0±1.67 | 0.6±0.08 | 0.3±0.31 |
