# Supplementary material for: Activation of cryptic xylose metabolism by a transcriptional activator Znf1 boosts up xylitol production in the engineered Saccharomyces cerevisiae lacking xylose suppressor BUD21 gene
Source: Microb Cell Fact. 2022 Mar 5;21:32. doi: 10.1186/s12934-022-01757-w (PMC8897867; doi:10.1186/s12934-022-01757-w)
Supplement: Supplementary file 2 — Additional file 2: List of plasmids, primers and strains used in this study. [file 12934_2022_1757_MOESM2_ESM.docx]

**Supplementary Table 2 List of plasmids, primers and strains used in this study**

| **Plasmid, Primer or Strain** | **Marker/ Sequence/ Genetic background** | **Reference** |
| --- | --- | --- |
| **Plasmid** | | |
| pRS316 | *URA3* marker under *TPI* promoter | (Jensen et al., 2019) |
| pLJ529-*ZNF1* | *URA3* marker, containing the native *ZNF1* gene of BY4742 under *TPI* promoter | (Jensen et al., 2019) |
| **Primer** | | |
| *ACT1* | F 5’-ATTATATGTTTAGAGGTTGCTGCTTTGG-3’  and R 5’- CAATTCGTTGTAGAAGGTATGATGCC-3’ | This study |
| *ADH1* | F 5’-TGGAAGATCGGTGACTACGCC-3’ and R 5’-CAGCACCGGAGATAGCAA-3’ | This study |
| *BUD21* | F 5’-GTGCTGTCACGGATAGACAAG-3’ and R 5’-CAACTCCTCAGGAAGCTCC-3’ | This study |
| *COX1* | F 5’-TCAGGTGCTGGTACAGGGTG-3’ and R 5’-TAACATTGTAATACCAGCAG-3’ | This study |
| *CYC3* | F 5’-ATGGACTTGCCCGTTGATCG-3’and R 5’-GCTGCACGTGGCTTTCATCT-3’ | This study |
| *ENO1* | F 5’-CACAGGTGGAACAAGCTGCC-3’ and R 5’-GGTCTGTCCTGTTGGATATA-3’ | This study |
| *FBA1* | F 5’-GCGTCATTGATCGAGTCAAG-3’ and R 5’-GGTTGCGCTGCATCAACATC-3’ | This study |
| *GAL2* | F 5’-GGCTGGGATACCGGTACTA-3’ and R 5’-CAGGATCCTCTGGTGACAC-3’ | This study |
| *GCY1* | F 5’- CGGTCAAGCCATCAAGGA-3’and R 5’- GTGATATCCACTGCACGAG-3’ | This study |
| *GDH2* | F 5’-CTGGTGTCAGCATAAGCGAT-3’ and R 5’-TTCAGCAAATCCTGAGAGGA-3’ | This study |
| *GLK1* | F 5’-ACGTCACATCTGACGACC-3’ and R 5’-AGAGAGGTCTGGTCTACAGG-3’ | This study |
| *GND1* | F 5’-GCCAGGTGGTTCTGAAGAA-3’ and R 5’-TTCTCTGTCCTTGACGGC-3’ | This study |
| *GPD1* | F 5’-GTGGGTGTTCGAAGAAGAG-3’ and R 5’-ACAGGAGATAGCTCTGACG-3’ | This study |
| *GPD2* | F 5’-GACTCTGCCGTGTCAATTG-3’ and R 5’-ATCAGGATCGGCCACTAGA-3’ | This study |
| *GPM1* | F 5’-CGGTGACTTACAAGGTAAGGAC-3’ and R 5’-GCCAGTATGGCAACAATCTG-3’ | This study |
| *GRE3* | F 5’-GCGCAGATGACGAGAAGA-3’ and R 5’-GTGGTACTGCCTGGATG-3’ | This study |
| *HXK1* | F 5’-AGGTCAATAGATGACCGCAA-3’ and R 5’-GATTACTACTGTTCCTGCTG-3’ | This study |
| *HXK2* | F 5’-CAACTGGTAAGGAATCCGG-3’ and R 5’-TGGCTCAGAGATACCTTGTG-3’ | This study |
| *HOR2 (GPP2)* | F 5’-TCTCGCATGGTTGGAGAAC-3’ and R 5’-CGTTGCACAGCTTAACTGC-3’ | This study |
| *HXT4* | F 5’-CGGTTGGGATACTGGTAC-3’ and R 5’-GTGTAGGTCTTGGTACCG-3’ | This study |
| *HXT7* | F 5’-GTGTTGGTGGTATTGCCG-3’ and R 5’-CAAGACAGCTTCGACTTCAGCTA-3’ | This study |
| *PFK1* | F 5’-CATCACCTTCTCAGGCTTA-3’ and R 5’-GAACGAGCAGTACCAATCA-3’ | This study |
| *PFK2* | F 5’-CCAAGACTCAAGACTGGAGA-3’and R 5’-ACCACCGGAAGTCATGAC-3’ | This study |
| *PDC1* | F 5’-TCATCACCACCTTCGGTG-3’ and R 5’-CAGTGAAGTCACCGTTACC-3’ | This study |
| *PGI1* | F 5’-GATTGAACTGGCCAAGGAG-3’ and R 5’-ATCGGTGATCTTCTTACCGG-3’ | This study |
| *PGK1* | F 5’-TTCTTGAACGACTGTGTCGG-3’ and R 5’-TACCGAAGGCATCGTTGATG-3’ | This study |

**Table 2 List of plasmids, primers and strains used in this study (Cont.)**

| **Plasmid, Primer or Strain** | **Marker/ Sequence/ Genetic background** | **Reference** |
| --- | --- | --- |
| *PYC1* | F 5’-ATGACAAATGCAGTAGCAAC-3’ and R 5’-GCTGACGAAGCATACGTCATA-3’ | This study |
| *PYK1* | F 5’-AGAACTGGTACCACCACC-3’and R 5’-AAGTCACCTCTGGCAACCA-3’ | This study |
| *RHR2 (GPP1)* | F 5’-TACGGTGAACACTCCATCG-3’ and R 5’-GACATCATTGGCGGTGATG-3’ | This study |
| *RKI1* | F 5’-GGAATTGGTAGTGGTAGCAC-3’ and R 5’-CACCGAAATCCGCATCGATA-3’ | This study |
| *RPE1* | F 5’-CTGCGTTCTTCGATTGTCAC-3’ and R 5’-AGCGACAATAACGTTGGC-3’ | This study |
| *SDH1* | F 5’-CGGGCTACAAGACTGCTTGT-3’ and R 5’-CTCCTTGGTCTGACCACCAA-3’ | This study |
| *SOL3* | F 5’-TGCCTTCAAGAGAGCTGTTC-3’ and R 5’-CGTGGCACCAAGCAACTC-3’ | This study |
| *SOL4* | F 5’-CATTAATCGGTGGCGGTG-3’ and R 5’-GCACACTAGGTAGTTCGC-3’ | This study |
| *SOR1* | F 5’-GTAACGGCTAGGAACACCA-3’ and R 5’-CCTCTTCATAACTGACGCC-3’ | This study |
| *SOR2* | F 5’-GTCATGAATCAAGCGGACAG-3’ and R 5’-ATAACTGACGCCTTCTGGC-3’ | This study |
| *TAL1* | F 5’-GCATGGTAAGACCACCGAA-3’ and R 5’-GCAACTGCTTGAACGAAGG-3’ | This study |
| *TDH1* | F 5’-CATGTCACCGACATGTCTGA-3’and R 5’-AGATCGATGTCGCTGTTGAC-3’ | This study |
| *TKL1* | F 5’-CTCTATCGAGGACTTGAGAC-3’ and R 5’-CCTCTTGTAAGCAACCATCC-3’ | This study |
| *TPI1* | F 5’-CTTGAAGGCTTCTGGTGC-3’ and R 5’-CTTCTTCCAAGACAGCGTTC-3’ | This study |
| *XKS1* | F 5’-CCGTAGCCATGTGGTTAGA-3’ and R 5’-GGCATCTGCCTCCTCTAA-3’ | This study |
| *XYL2* | F 5’-GGGCAGATACAACCTGGA-3’ and R 5’-CACCAAAGACACTGGCTAC-3’ | This study |
| *YDL124W* | F 5’- AAGGCACTTAGCCTCACC-3’ and R 5’- CATGTCCTTCCAGGCTTC-3’ | This study |
| *YJR096W* | F 5’- GCAAGAAGCGGTTGATGAAG-3’ and R 5’- GACCTGGATTACGGTCCA-3’ | This study |
| *YPR1* | F 5’- TACGGAACAACGTGATCCG-3’ and R 5’- GTAGCTGGTACCACCTTG-3’ | This study |
| *ZNF1* | F 5’-AGGCACTAATTGATCAGTGTCTGC-3’  and R 5’-GCAGAAACTGGATAACTGTATCC-3’ | This study |
| *ZWF1* | F 5’-CCGATGACTCTAAGGTCGAA-3’ and R 5’-GATCTGAATCTAACTTACGC-3’ | This study |
| **Strain** | | |
| BY4742 | *MATα his3* Δ1; *leu2* Δ0; *lys2* Δ0; *ura3* Δ0 | (Brachmannet,1998) |
| *znf1*Δ | *MATα his3* Δ1; *leu2* Δ0; *lys2* Δ0; *ura3* Δ0; *yfl052w*::*kanMX4* | Open Biosystems |
| *bud21*Δ | *MATα his3* Δ1; *leu2* Δ0; *lys2* Δ0; *ura3* Δ0; *yor078w*::*kanMX4* | Open Biosystems |
| BY4742+pRS316 | BY4742, pRS316 (*URA3*) | This study |
| *znf1*Δ+pRS316 | *znf1*Δ, pRS316 (*URA3*) | This study |
| *bud21*Δ+pRS316 | *bud21*Δ, pRS316 (*URA3*) | This study |
| BY4742+pLJ529-*ZNF1* | BY4742, pLJ529-*ZNF1* (*URA3*) | This study |
| *znf1*Δ+pLJ529-*ZNF1* | *znf1*Δ, pLJ529-*ZNF1* (*URA3*) | This study |
| *bud21*Δ+pLJ529-*ZNF1* | *bud21*Δ, pLJ529-*ZNF1* (*URA3*) | This study |

**Table 2 List of plasmids, primers and strains used in this study (Cont.)**

| **Plasmid, Primer or Strain** | **Marker/ Sequence/ Genetic background** | **Reference** |
| --- | --- | --- |
| *ZNF1*-OE | BY4742, Genome integration of 1 copy of native *ZNF1* of BY4742 under *TEF1* promoter | (Samakkarn et al., 2021) |
